# MKS-NPHP module proteins control ciliary shedding at the transition zone

**DOI:** 10.1371/journal.pbio.3000640

**Published:** 2020-03-12

**Authors:** Delphine Gogendeau, Michel Lemullois, Pierrick Le Borgne, Manon Castelli, Anne Aubusson-Fleury, Olivier Arnaiz, Jean Cohen, Christine Vesque, Sylvie Schneider-Maunoury, Khaled Bouhouche, France Koll, Anne-Marie Tassin

**Affiliations:** 1 Université Paris-Saclay, CEA, CNRS, Institute for Integrative Biology of the Cell (I2BC), Gif-sur-Yvette, France; 2 Sorbonne Université, CNRS UMR7622, INSERM U1156, Developmental Biology Laboratory—Institut de Biologie Paris-Seine (IBPS), Paris, France; Rheinische Friedrich-Wilhelms-Universitat Bonn, GERMANY

## Abstract

Ciliary shedding occurs from unicellular organisms to metazoans. Although required during the cell cycle and during neurogenesis, the process remains poorly understood. In all cellular models, this phenomenon occurs distal to the transition zone (TZ), suggesting conserved molecular mechanisms. The TZ module proteins (Meckel Gruber syndrome [MKS]/Nephronophtysis [NPHP]/Centrosomal protein of 290 kDa [CEP290]/Retinitis pigmentosa GTPase regulator-Interacting Protein 1-Like Protein [RPGRIP1L]) are known to cooperate to establish TZ formation and function. To determine whether they control deciliation, we studied the function of 5 of them (Transmembrane protein 107 [TMEM107], Transmembrane protein 216 [TMEM216], CEP290, RPGRIP1L, and NPHP4) in *Paramecium*. All proteins are recruited to the TZ of growing cilia and localize with 9-fold symmetry at the level of the most distal part of the TZ. We demonstrate that depletion of the MKS2/TMEM216 and TMEM107 proteins induces constant deciliation of some cilia, while depletion of either NPHP4, CEP290, or RPGRIP1L prevents Ca2+/EtOH deciliation. Our results constitute the first evidence for a role of conserved TZ proteins in deciliation and open new directions for understanding motile cilia physiology.

## Abstract 2

Functional analysis and subcellular localisation of the conserved transition zone proteins in the ciliate Paramecium tetraurelia demonstrates their involvement in the ciliary shedding process, opening new avenues fir understanding the molecular mechanism of deciliation.

## Introduction

Cilia are conserved cell appendages endowed with motility and sensory functions acting as “cellular antennae.” The cilia that emanate from the surface of most cells in multicellular organisms are key organelles in numerous developmental and physiological processes [1]. Their architecture consists of 3 structural regions: basal body (BB) at their proximal part, the axoneme composed of 9 microtubule doublets covered by the ciliary membrane at the distal part and in between, and the transition zone (TZ). The TZ is characterized by (i) Y-shaped linkers connecting the microtubule doublets of the axoneme to the ciliary membrane, the so-called Y-links, and (ii) the transition fibers. These two structures are thought to function together as a gate [2], ensuring a specific ciliary composition. Genetic studies along with protein-protein interaction data and proteomics analyses led to the identification of various components of TZ [3,4,5,6,7,8] (for review see [2,9]). Some of them are assembled in two distinct protein complexes, defined as the Meckel Gruber syndrome (MKS) and the Nephronophtysis (NPHP) modules, which cooperate in biogenesis and function of the TZ [3]. Centrosomal protein of 290 kDa (CEP290) and Retinitis pigmentosa GTPase regulator- Interacting Protein 1-Like Protein (RPGRIP1L) play a crucial function in TZ assembly [10,11,12,13], where they have been proposed to be associated with the Y-links [14]. Importantly, many genes encoding TZ proteins are mutated in human ciliopathies [15].

While much is known about the multistep processes involved in ciliogenesis (from BB duplication to cilia elongation), ciliary shedding (also called deciliation, flagellar excision, or autotomy) is still poorly understood. Deciliation is different from cilia resorption/involution and is characterized by the shedding of cilia or flagella at the distal end of the TZ [16,17,18,19]. Deciliation occurs in unicellular organisms as well as in vertebrate multiciliated epithelia. In the oviduct, cilia autotomy has been observed both in birds and in mammals during the luteal phase of the menstrual cycle [20,21,22,23]. This process is more pronounced after progesterone therapy [24]. Smoke and nitrogen dioxide inhalation [25] were shown to induce deciliation in the upper airway. Disorganization of the ciliary necklace, and in some cases its absence, have been observed [26,27,28] during upper airway infection, suggesting a strong effect of bacterial infection on the TZ leading to subsequent loss of cilia. Although deciliation in metazoans has been mostly studied in motile multiciliated cells, the shedding of primary cilia during the cell cycle was recently demonstrated by long-term video recording of cultured inner medullary collecting duct 3 (IMCD3) cells [29]. Similarly, ciliary shedding seems to occur during neurogenesis to allow the newly born neuron to migrate out of the ventricle [30].

Mechanisms of deciliation have been studied using the unicellular organism *Chlamydomonas* [19,31]. In this organism, deflagellation is triggered by an increase in intracellular calcium near the base of the flagella [32,33,34] and involves microtubule-severing proteins such as katanin [35,36,37]. Interestingly, proteins involved in this process—such as Flagellar automy 1p (Fa1p), Flagellar automy 2p (Fa2p) belonging to the Never in mitosis A related kinase (Nek) kinase family, and Flagellar associated protein 16 (FAP16)—have been shown to localize at the TZ [37,38,39].

To evaluate a possible involvement of the conserved MKS/NPHP proteins in the deciliation process of motile cilia, we used *Paramecium* to study 5 proteins: two belonging to the MKS module (MKS2/Transmembrane protein 216 [TMEM216] and TMEM107), one belonging to the NPHP module (NPHP4), and the regulators of TZ assembly CEP290 and RPGRIP1L.

We show using super-resolution microscopy and transmission electron microscopy (TEM) that all 5 proteins localize with 9-fold symmetry at the distal part of the TZ, which is consistent with their localization in non-motile and motile cilia of various organisms [9,40]. In *Paramecium*, the TZ displays 3 plates, from proximal to distal: the terminal plate, the intermediate plate, and the axosomal plate, which appears adjacent to the ciliary necklace [41]. We show using functional RNA interference (RNAi) studies that the depletion of TZ proteins induces only minor defects in TZ structure that do not impair ciliary growth [42]. Most interestingly, the depletion of any of these proteins impacts the deciliation process. While the depletion of the two MKS protein modules induces constant ciliary autotomy, the depletion of either NPHP4, CEP290, or RPGRIP1L increases resistance to deciliation. Our results constitute the first evidence for a role of these TZ proteins in deciliation and open new directions for understanding cilia physiology.

## Results

**Fig 1:**
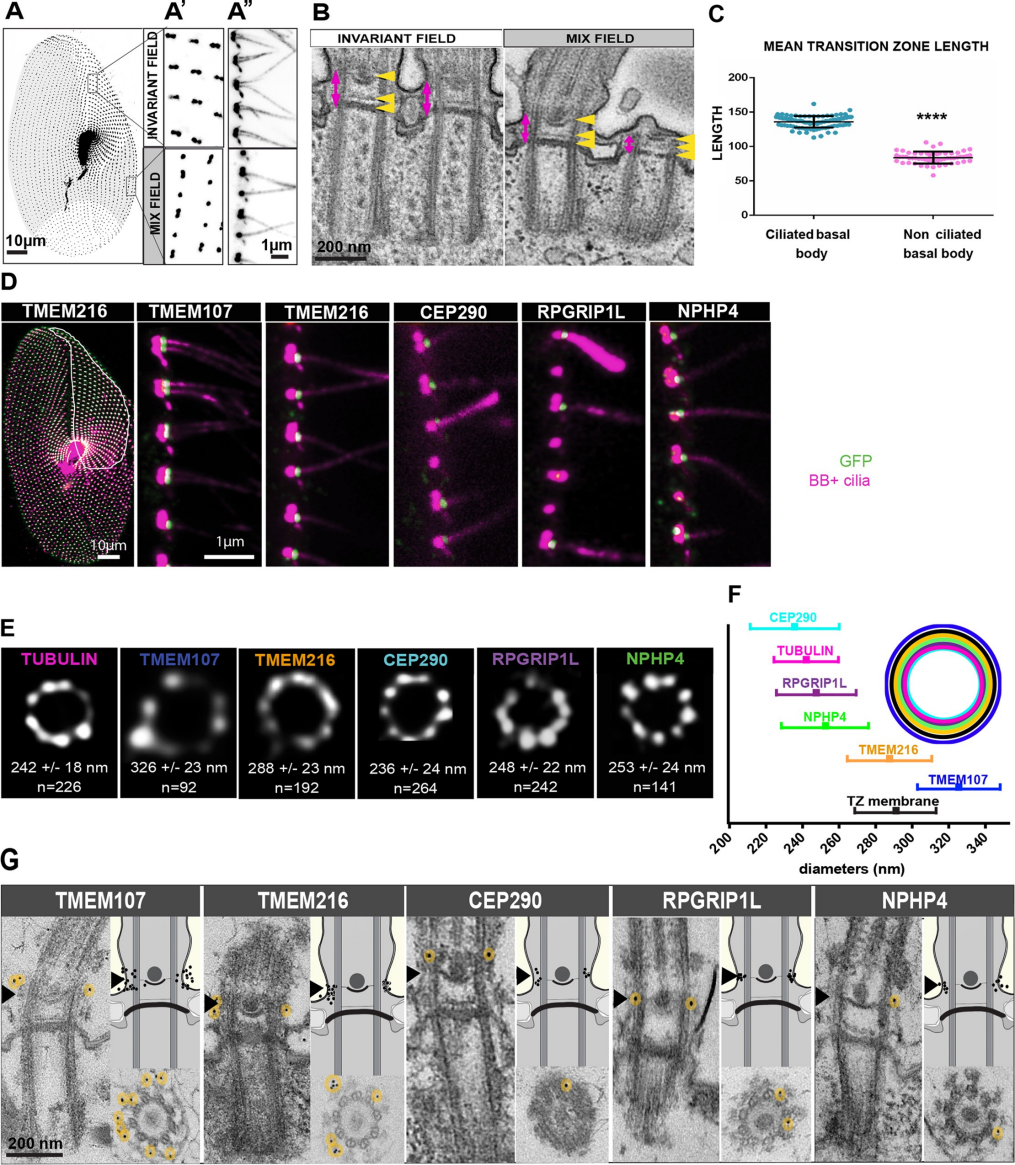
Localization of TZ proteins in *Paramecium*. (A–C) Ciliation status in *Paramecium*. (A) Paramecia labelled by the monoclonal anti-glutamylated tubulin ID5 (decorating BB and cilia). Paramecium cortex presents different regions in which the BB pattern differs. BBs are organized in doublets in the invariant field region. The mixed field, highlighted here in light grey, presents interspersed BB singlets and doublets. The posterior region, in white, only possesses singlet BBs. A′ is a magnification of the surface view. A′′ is a transverse section showing that in the invariant field each BB of the doublet is ciliated, while in the mixed field only the posterior BB bears a cilium. Bars = 10 μm and 1 μm, respectively. (B) EM images showing a longitudinal section of two-BB units in the invariant field (left) and mixed field (right). The TZ (magenta arrow) is characterized by the presence of 3 successive layers indicated by yellow arrowheads. From bottom to top: the terminal plate, the intermediate plate, and the axosomal plate. Note that the unciliated BB in the mixed field shows a reduced TZ compared to ciliated ones. Bar = 200 nm. (C) Graph showing the mean length of the TZ of ciliated and unciliated basal bodies (>4 replicates). Ciliated BB counted: 100, nonciliated BB counted: 56. Ciliated TZ length: 136 nm and unciliated TZ length: 84 nm. Unpaired two-sided *t* test, ****p < 0.0001. Source data can be found in S1 Data. (D–G) Localization of *Paramecium* TZ proteins. (D) Paramecia expressing different TZ proteins fused with GFP. Cells were permeabilized before proceeding to immunostaining by ID5 (decorating BB and cilia in magenta) and a polyclonal anti-GFP (in green). Left panel: surface view of a Paramecium expressing TMEM216-GFP. Ciliated basal bodies of the invariant field (encircled in white) are stained by both GFP antibodies and ID5. In the other part of the cells, some basal bodies are labelled only by ID5. Right panels: confocal Z projections of ciliary rows, at the cell margin from TZ protein transformants. TMEM107-GFP, TMEM216-GFP, CEP290-GFP, and RPGRIP1L-GFP are localized only on the distal part of ciliated BBs. In addition, NPHP4-GFP can be observed at the BB proximal part. Note that ID5 antibodies better recognize short cilia. Bars = 10 μm and 1 μm. (E) Representative STED images revealing distinct localization patterns of several GFP-tagged TZ proteins. Cells were labelled with anti-GFP or ID5 (tubulin). A single ring differing in diameter is observed according to the observed protein. The mean diameters (distance between intensity maxima) and the number of BBs analyzed are given beneath each image, 2 replicates. (F) Graph (left) showing the mean diameter ± SD of each toroid labeled by each GFP tagged TZ protein (see B). Top right, schema representing the relative position of all toroids with respect to the position of tubulin and the ciliary membrane. Each TZ protein is shown in a different color. Source data can be found in S1 Data. (G) Representative EM images of the immunolocalization of the different GFP fusion proteins revealed by anti-GFP antibodies. Left panels: longitudinal views. Lower right panels: transverse sections of BBs at the level of the axosomal plate. Upper right panels: BB diagrams recapitulating the localization of gold beads. All these proteins, although they occupy different diameters in transverse views, are localized at the level of the axosomal plate indicated by a black arrowhead. TMEM107: 35 gold beads on 16 BB; TMEM216: 36 gold beads on 21 BB; CEP290: 11 gold beads on 10 BB; RPGRIP1L: 24 gold beads on 18 BB; NPHP4: 14 gold beads on 8 BB. Gold beads are highlighted in yellow. Bars: 200 nm. BB, basal body; CEP290, centrosomal protein of 290 kDa; EM, electron microscopy; GFP, green fluorescent protein; NPHP4, Nephronophtysis 4; RPGRIP1L, Retinitis pigmentosa GTPase regulator-Interacting Protein 1-Like Protein; STED, Stimulated Emission Depletion; TMEM216, Transmembrane protein 216; TZ, transition zone.

We performed this study in *Paramecium tetraurelia*, a unicellular organism that bears at its surface about 4,000 motile cilia. Paramecia display a precise BB and cilia organization [43,44] (for review see [45], Fig 1A). In the anterior part of the cell, the invariant field shows cortical units with two ciliated BBs, while the posterior part displays units with a single BB. In between, the mixed field exhibits units with either a single or two BBs. In the latter case, both BBs are anchored at the cell surface, but only the posterior one is ciliated (Fig 1B). As in *Tetrahymena* [46], single BBs can be either ciliated or not, according to the cell cycle stage ([47]). Interestingly, ultrastructural analysis of the TZ of unciliated or ciliated BBs reveals that they have a distinct morphology. The ciliated BBs have longer TZs, suggesting that the process of structural modification may occur during ciliation (Fig 1B and 1C, [44,45]).

Bioinformatic analyses showed that CEP290 and RPGRIP1L as well as most MKS and NPHP module proteins are conserved in *Paramecium* (Table 1; [11,48]). We focused on 5 of them: two belonging to the MKS module and interacting together [43] (TMEM216/MKS2 and TMEM107), one representative of the NPHP complex (NPHP4), and the two pivotal proteins CEP290/NPHP6 and RPGRIP1L/MKS5/NPHP8. We will refer to them as a whole as “TZ proteins.” As a result of whole genome duplications during evolution of this lineage [49], several paralogs of each protein are encoded in the *P*. *tetraurelia* genome (See S1 Table).

**Table 1:** Conservation of TZ proteins in *Paramecium*.

	NAME	PRESENCE IN *PARAMECIUM*
**MKS MODULE**	MKS1	Yes
	MKS2/TMEM216	Yes
	MKS3/TMEM67/Meckelin	Yes
	Ahi1	Yes
	B9D1	Yes
	B9D2	Yes
	Tectonic (1,2,3)	Yes
	TMEM17	Yes
	TMEM107	Yes
	TMEM218	-
	TMEM231	Yes
	TMEM237	-
**NPHP MODULE**	NPHP1	-
	NPHP4/POC10	Yes
	NPHP5/IQCB1	-
**OTHERS**	NPHP3	-
	CEP290/NPHP6/MKS4	Yes
	RPGRIP1L/NPHP8/MKS5	Yes

In *Paramecium*, most of the TZ proteins are conserved.

**Abbreviations:** CEP290, Centrosomal protein of 290 kDa; MKS, Meckel Gruber syndrome; NPHP, Nephronophtysis; RPGRIP1L, Retinitis pigmentosa GTPase regulator-Interacting Protein 1-Like Protein; TMEM, Transmembrane protein; TZ, transition zone

### TZ proteins are recruited at the axosomal plate at the onset of ciliogenesis

We assessed whether the candidate proteins localized at the TZ, like their counterparts in other organisms, by expressing one green fluorescent protein (GFP)-tagged paralog of each protein. After detergent extraction and fixation, microscopic analysis performed on each GFP-transformed cell line detected GFP fluorescence only on ciliated BBs (Fig 1D and S1A Fig). Fluorescence localized between the BB and the cilium, which is compatible with TZ labelling. In addition to this common localization, NPHP4-GFP and CEP290-GFP were also present on the proximal part of all BBs (Fig 1D, S1A Fig). Additional cortical staining associated with all BB for TMEM216-GFP was observed on living or fixed cells after mild detergent extraction (S1A Fig). In subsequent analyses, we focused on the putative TZ labelling of each of the 5 proteins.

We used super-resolution microscopy (Stimulated Emission Depletion [STED] microscopy) to increase localization accuracy (Fig 1E and 1F). All proteins were organized with a 9-fold symmetry. Quantification of the mean diameters of the rings revealed that both CEP290-GFP and RPGRIP1L-GFP localized most centrally, close to the microtubules, whereas NPHP4-GFP was detected outside the ring of microtubule doublets. TMEM216-GFP and TMEM107-GFP were found more externally (Fig 1E and 1F). Immuno-electron microscopy confirmed these observations and suggested that TMEM216-GFP as well as TMEM107-GFP are more closely localized to the TZ ciliary membrane whereas CEP290-GFP and RPGRIP1L-GFP are localized close to the tubulin scaffold. Interestingly, a clear restriction of all these proteins to the axosomal plate (arrowheads on Fig 1G) is found. This localization in *Paramecium* corresponds to the localization of the ciliary necklace [41] and the Y-links, which connect transition fibers to the ciliary membrane.

To decipher the timing of recruitment of proteins at the TZ, we analyzed their localization during ciliary growth (see Materials and Methods). Each protein is recruited to the TZ as soon as the growing cilium is detected by the antibodies (S1B Fig).

Altogether, these results indicate that all 5 proteins are localized at the TZ, as in other organisms [3,9,11,14,40,42,50]. In addition, the protein recruitment at the TZ strongly suggests that molecular maturation of the TZ accompanies structural maturation.

### Depletion of TZ proteins affects proliferation rate and swimming velocity

To ascertain the function of these TZ proteins, all paralogs of each protein family were depleted concomitantly by inactivating all the corresponding genes using the feeding method (TZ^RNAi^) [51,52]. The efficiency of silencing was tested by analyzing either the mRNA level or by controlling the effective depletion of the GFP-tagged protein by quantification of the BB fluorescence (S2 Fig, Materials and Methods). Within the first 24 h, depleted cells showed abnormal swimming behavior associated with a slower growth rate (S3A Fig). These phenotypes were stably maintained for several days in the RNAi media except for TMEM216/MKS2-depleted cells, which stopped dividing after 72 h.

To better characterize the motility defects in silenced cells, we video-recorded paramecia. Control cells had an average swimming velocity of 683 μm per second (S3B Fig). Depletion of TZ proteins resulted in a severe reduction (by about one-third) of this velocity (S3B Fig). Such a reduction has already been observed in paramecia after depleting Basal body up-regulated protein 22 (Bug22) [53], dynein axonemal heavy chain 9 (DNAH9), or C11orf70, these last two proteins being required for dynein arm assembly [54,55].

### Depletion of TMEM107 and TMEM216 leads to an accumulation of stretches of short cilia

**Fig 2:**
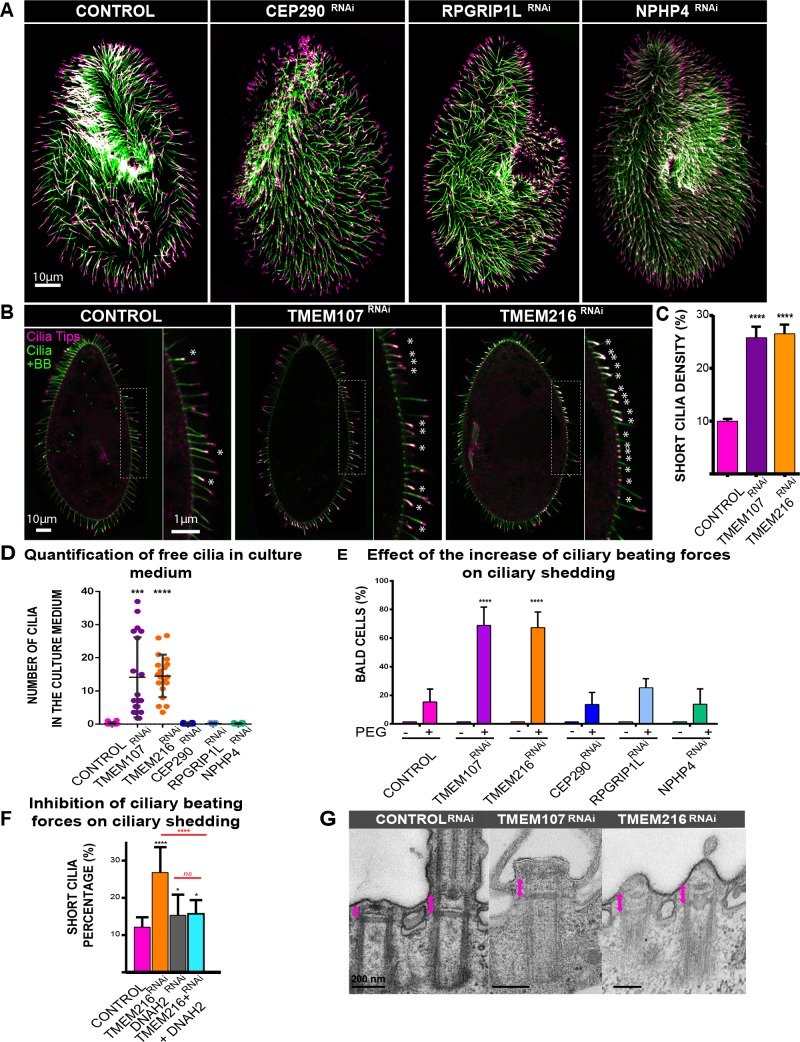
Ciliary pattern of paramecia depleted for TZ proteins. (A) Ciliary pattern of paramecia treated with control RNAi or TZ^RNAi^ (CEP290, RPGRIP1L, and NPHP4). Cells were immunostained by the monoclonal anti-mono-glycylated tubulin TAP952 (magenta, cilia tip labelling) and the polyclonal anti-poly-glutamylated tubulin (polyE) antibodies (green, decorating BB and cilia). These TZ-protein–depleted paramecia display a ciliary pattern similar to that of control paramecia. (B) Control, TMEM107-depleted, and TMEM216-depleted paramecia stained for cilia using TAP952 (magenta) and the poly-E tubulin (green). Control paramecia show the usual ciliary pattern with long cilia (10 μm) and a few short growing cilia indicated by asterisks. A large increase in short (about 4 μm) or tiny cilia is observed in TMEM107- and TMEM216-depleted cells. Bar = 10 μm. (C) Bar plot showing the mean percentage of short cilia in Control (*n* = 60 cells, 3 independent replicates), TMEM107 (*n* = 50 cells, 3 independent replicates), and TMEM216-depleted (*n* = 42 cells, 3 independent replicates) cells. Error bars show the SEM. Statistical significance was assessed by an unpaired *t* test, two-sided *p* < 0.0001****. Source data can be found in S2 Data. (D–G) Ciliary shedding in TMEM107- or TMEM216-depleted cells. (D) Quantification of free cilia in culture medium: dot plot showing the number of free cilia found in the culture medium (about 10 microscope fields were analyzed per experiment; see Materials and Methods). Two independent replicates. Cilia were labelled using ID5 and poly-E antibodies. Error bars represent the standard deviation. Statistical significance was assessed by an unpaired *t* test. ***p = 0.0004, ****p < 0.0001. Source data can be found in S2 Data. (E) Effect of the increase of ciliary beating forces on ciliary shedding quantification of the mean number of bald cells (<25% cilia per cell) after 1 h in 10% PEG for control (*n* = 410 cells, 8 independent replicates), TMEM107^RNAi^ (*n* = 316, 5 independent replicates), TMEM216^RNAi^ (*n* = 303 cells, 5 independent replicates), CEP290^RNAi^ (*n* = 291, 4 independent replicates), RPGRIP1L^RNAi^ (*n* = 204, 3 independent replicates), and NPHP4^RNAi^ (*n* = 196, 3 independent replicates). Errors bars represent the standard deviation. Statistical significance was assessed by unpaired two-sided χ^2^ test, ****p < 0.0001. Confidence interval 95% source data can be found in S2 Data. (F) Effect of inhibition of ciliary beating on ciliary shedding; quantification of the mean percentage of short cilia in Control (*n* = 60 cells, 2 independent replicates), TMEM216^RNAi^ (*n* = 42 cells, 2 independent replicates), DNAH2^RNAi^ (*n* = 15 cells, 2 independent replicates), and TMEM216-DNAH2^RNAi^ (*n* = 16 cells, 2 independent replicates) paramecia. DNAH2^RNAi^ cells present a percentage of short cilia slightly higher than the controls. Impairing ciliary beating of TMEM216^RNAi^ cells by DNAH2 decreases this percentage. Statistical significance was assessed by unpaired two-sided *t* test. *p*-Values: *p = 0.0306, ****p < 0.0001. Source data can be found in S2 Data. (G) EM images of ciliary defects induced by TMEM107^RNAi^ and TMEM216^RNAi^. Control^RNAi^ basal bodies showing a two-BB unit, with one unciliated and one ciliated BB. The length of the TZ is indicated by a red arrow. Cilia are either severed at the level of the axosomal plate as shown in TMEM107^RNAi^ or have been severed and are in a regrowth process as in TMEM216^RNAi^. Note that the length of the TZ corresponds to the length of TZ of ciliated BB. BB, basal body; CEP290, centrosomal protein of 290 kDa; DNAH2, dynein axonemal heavy chain 2; NPHP4, Nephronophtysis 4; ns, nonsignificant; PEG, Polyethylene glycol; polyE, anti-poly-glutamylated tubulin; RNAi, RNA interference; RPGRIP1L, Retinitis pigmentosa GTPase regulator-Interacting Protein 1-Like Protein; TMEM216, Transmembrane protein 216; TZ, transition zone.

To determine whether the swimming alterations could be due to defects in ciliation or in BB organization, we analyzed their patterns by immunolabelling. In all silenced cells, the BB pattern was normal (S4 Fig), indicating that TZ protein depletion did not affect BB duplication or anchoring processes. Cep290^RNAi^, RPGRIP1L^RNAi^, and NPHP4^RNAi^ cells displayed a ciliary pattern essentially identical to the control cells (Fig 2A). In contrast, interphasic TMEM107^RNAi^ or TMEM216^RNAi^ cells displayed an accumulation of stretches of short cilia, measuring about 4 μm (Fig 2B). Such stretches of short cilia were never observed in control cells. Quantification of these short cilia (Fig 2C) confirmed a dramatic increase in their number (control cells [9.92% ± 0.5%], TMEM107^RNAi^ [25.8% ± 2%], and TMEM216^RNAi^ [26.55% ± 1.7%]). Several hypotheses may explain this phenotype such as slow ciliary growth, ciliary involution, or constant ciliary breakage (i.e., some cilia deciliate) followed by ciliary regrowth.

### TMEM107^RNAi^ and TMEM216^RNAi^ induce constant ciliary breakage

To first assess whether the ciliary phenotype observed in TMEM107^RNAi^ or TMEM216^RNAi^ cells could be caused by constant cilia breakage/regeneration, we looked for the presence of free detached cilia in the culture medium (see Materials and Methods). Although no cilia were found in the medium of either control, CEP290^RNAi^, RPGRIP1L^RNAi^, or NPHP4^RNAi^ cells, numerous cilia were detected in TMEM107^RNAi^ (about 15 cilia per field, 2 independent replicates) or TMEM216^RNAi^ (about 12 cilia, 2 independent replicates) (Fig 2D). These results support the hypothesis that the short cilia observed after depletion of TMEM107 or TMEM216 result from constant ciliary breakage followed by reciliation.

### Modification of the ciliary beating force influences ciliary shedding in TMEM107- and TMEM216-depleted cells

We hypothesized that the depletion of TMEM107 or TMEM216 weakened the cilia, which consequently did not resist the forces exerted to sustain ciliary beating or bending. One might expect that, in this situation, an increase of ciliary beating forces would intensify the deciliation phenotype while a decrease would alleviate it. To test this hypothesis, we first increased the standard-medium viscosity, known to induce an elevation of the ciliary force. After culture in 10% Polyethylene glycol (PEG)-containing medium, the ciliation pattern of TMEM107^RNAi^, TMEM216^RNAi^, or control cells was analyzed by immunofluorescence (IF). Number of bald cells (defined as less than 25% remaining cilia) was counted (Fig 2E). In contrast to the modest increase in cell number that became bald in control, CEP290^RNAi^, RPGRIP1L^RNAi^, and NPHP4 ^RNAi^ cells (17%, 15%, 21%, and 14%, respectively), there was a dramatic increase in this cell number in TMEM107^RNAi^ (71%) and TMEM216^RNAi^ (73%) conditions.

Conversely, we blocked the ciliary beating in TMEM216^RNAi^ cells by inactivating the gene encoding DNAH2, required for ciliary beating [56]. As expected, the depletion of DNAH2 alone as well as co-depletion of DNAH2/TMEM216 led to a complete immobilization of the cells within 48 h. As predicted, the co-depletion of TMEM216 and DNAH2 reduced ciliary breakage from 27% in TMEM216^RNAi^ to 15% (Fig 2F). Moreover, bald cells were never observed in the co-depletion conditions. DNAH2 depletion alone induces a slight increase in the percentage of short cilia compared to control cells (15% versus 12%, Fig 2F).

Altogether, these results demonstrate that TMEM107 and TMEM216 may reduce ciliary breakage by the forces generated during ciliary beating.

### Cilia shedding occurs at the distal part of the TZ, precisely at the axosomal plate level

To determine the site of cilia breakage in TMEM107^RNAi^ and TMEM216^RNAi^ cells, electron microscopy experiments were carried out. Control cells displayed the usual pattern of cortical two-BB units showing a ciliated and an unciliated BB (see Fig 1B). In cells depleted for TMEM107 or TMEM216, most of the cilia showed a normal morphology, as expected from the IF staining. Nevertheless, BBs harboring an extended severed TZ above the axosomal plate (Fig 2G TMEM107^RNAi^, S5 Fig) were seen typical of deciliated ones [57]. Interestingly, two-BB units with 2 short-growing cilia, characterized by the presence of electron dense material and extended TZ, were also observed (Fig 2G, TMEM216^RNAi^, S5 Fig). Such a situation is never observed in control cells. Two-BB units, either in the invariant field or in the mixed field (Fig 1), never grow their cilia simultaneously. Therefore, this feature suggests that at least one of them has been broken. Altogether, these results are in favor of a breaking point just above the distal end of the TZ, at the level of the axosomal plate.

### TMEM216 depletion affects ciliary and membrane fusion gene expression

**Fig 3:**
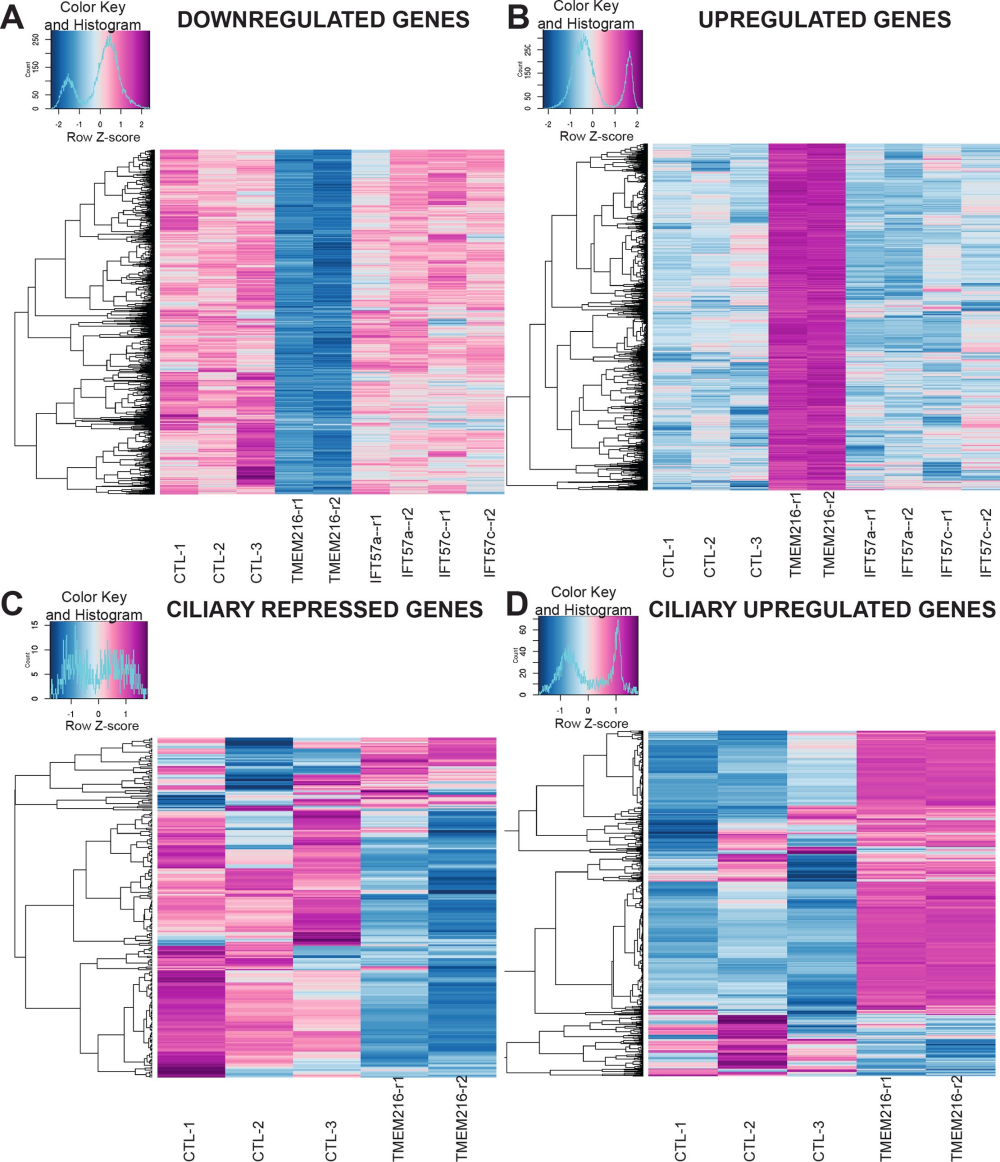
Transcriptomic analysis of TMEM216-depleted cells. The heatmaps were generated by the “heatmaps.2” R function using the log2 normalized gene expression levels in the different conditions. The color keys are presented at the top left corner of each heatmap and show a color variation from dark blue to dark red according to the gene expression level. (A–B) Each line represents the expression level variation for one down-regulated gene (A) or up-regulated gene (B) when TMEM216 is depleted. The differential expression level of the genes between control biological replicates (first three columns) and TMEM216^RNAi^ biological replicates (fourth and fifth column) is highlighted by the color variation. It is remarkable that these genes are not affected in IFT57-depleted cells (last four columns corresponding to the 4 biological replicates). (C–D) Comparison of the expression of genes differentially expressed (repressed [C] or up-regulated [D]) during the reciliation process [59] (called ciliary genes) with their expression in TMEM216^RNAi^ and control cells. In C, the vast majority of the ciliary repressed genes are also repressed when TMEM216 is depleted. Likewise, the ciliary up-regulated genes are up-regulated in TMEM216^RNAi^. IFT57, intraflagellar transport 57; RNAi, RNA interference; TMEM216, Transmembrane protein 216.

Ciliary regeneration following the deciliation process is correlated with a modification of the ciliary gene expression profile [58,59]. We expected that the depletion of TMEM107 or TMEM216 would also modify ciliary gene expression. We thus undertook a transcriptomic analysis of TMEM216-depleted paramecia by RNA deep sequencing. A total of 4,295 genes present a significant modification of their expression profile compared to controls (fold change of ± 2, S2 Table). The specific effect of silencing *TMEM216* on the transcriptome was confirmed by comparison with the transcriptome of intraflagellar transport 57 (IFT57^RNAi^) cells characterized by total disappearance of cilia [60]. The two transcriptomes showed no overlap (Fig 3A and 3B). Concerning TMEM216^RNAi^, one-fourth of the deregulated genes (1,079/4,295) correspond to genes identified as potential ciliary genes by transcriptomic, proteomic [58,61], and comparative genomic analyses (see S2 Table, sheets 2 and 3). This corresponds to an enrichment of ciliary genes compared to their representation in the complete genome (4,585/39,642). Remarkably, 535 genes identified in our RNA sequencing screen are also differentially expressed during the reciliation process, 95% of them (507) being up- or down-regulated in the same direction (426 genes are up-regulated both in response to TMEM216 ^RNAi^ and during reciliation and 81 are down-regulated, Fig 3C and 3D). These results suggest that the depletion of TMEM216 mimics a reciliation process as expected, a phenotype completely different from the IFT57^RNAi^ phenotype [60].

We specifically searched for different genes involved in ciliogenesis processes after TMEM216^RNAi^. Except for the down-regulation of the gene encoding the *Paramecium B9D2* orthologs or *IFT46* (fold change 0.48, *p* = 3.54 × 10^−4^, fold change 0.21, *p* = 3.8 × 10^−3^, respectively, S2 Table), the expression of genes belonging to the *MKS*, *NPHP*, *BBS*, or *IFT* complexes identified in *Paramecium* was not affected. Expression of a *VPS4* homolog and of a gene belonging to the *SNF7* family, both encoding proteins involved in membrane fusion and associated with the TZ in *Chlamydomonas* and mammalian cells [7,62], were significantly up-regulated (fold change 14.6, *p* = 3.17 × 10^−16^ and fold change 21.8, *p* = 8 × 10^−11^, respectively). In addition, the expression of another gene, *NSF*, whose encoded protein is involved in membrane fusion, is also greatly up-regulated (fold change 40.59, *p* = 4.79 × 10^−58^). These results suggest that these proteins may act to remodel the cell membrane after axoneme severing. In contrast, microtubule-severing proteins, such as katanin or spastin, are not differentially expressed after TMEM216 depletion or chemical deciliation.

### Depletion of CEP290, RPGRIP1L, or NPHP4 affects the deciliation process

**Fig 4:**
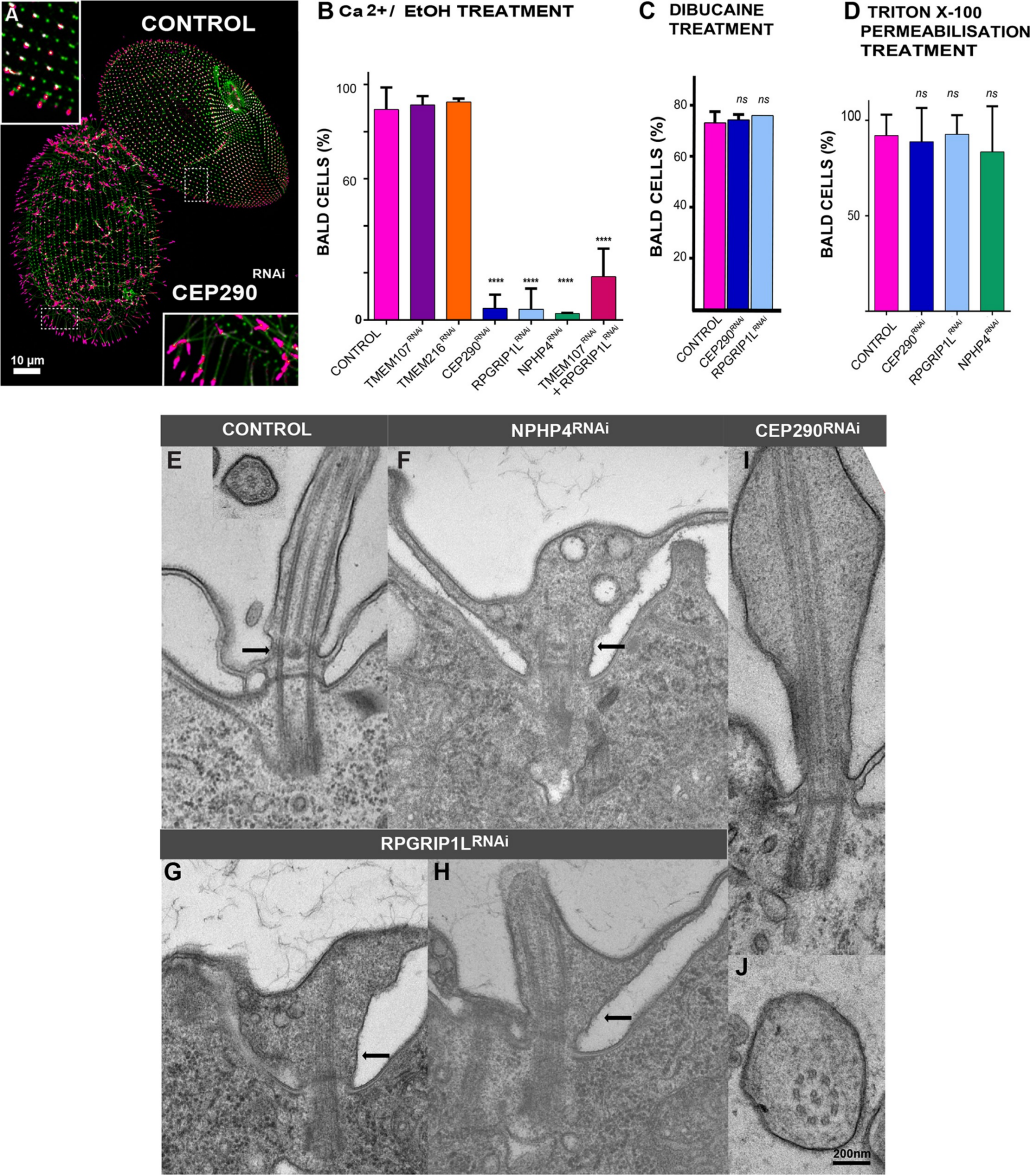
Depletion of CEP290, NPHP4, or RPGRIP1L affects deciliation ability and ciliary shape. (A–D) Depletion of CEP290, NPHP4, or RPGRIP1L affects deciliation ability. (A) Control and CEP290-depleted cells were simultaneously submitted to deciliation treatment (5% EtOH, 1 mM Ca2+) prior to labelling cilia (TAP952 in magenta, polyE in green). Control cells were previously marked by India ink ingestion allowing their identification. Cep290^RNAi^ cells bear cilia, whereas control cells are completely bald. Bar = 10 μm. (B) Bar plot showing the mean percentages of bald cells (less than 25% of cilia) after deciliation for control (*n* = 421 cells), TMEM107^RNAi^ (*n* = 290 cells), TMEM216^RNAi^ (*n* = 258 cells), CEP290^RNAi^ (*n* = 98 cells), RPGRIP1L^RNAi^ (*n* = 302 cells), NPHP4^RNAi^ (*n* = 82 cells), and the double TMEM107/RPGRIP1L^RNAi^ (*n* = 141 cells; error bars represent the standard deviation); >3 independent replicates per condition. Statistical significance was assessed by unpaired χ^2^ test. ****Two-sided *p* < 0.0001. Source data can be found in S3 Data. (C) Bar plot showing the mean percentages of bald cells (less than 25% of cilia) for control (*n* = 53 cells), CEP290^RNAi^ (*n* = 47 cells), and RPGRIP1L^RNAi^ (*n* = 68 cells) after deciliation with 5 mM dibucaine. Error bars represent the standard deviation. Three replicates per condition. Statistical significance was assessed by unpaired χ^2^ test two-sided *p*-value. Source data can be found in S3 Data. (D) Bar plot showing the mean percentages of bald cells (less than 25% of cilia) for control (*n* = 222 cells), CEP290^RNAi^ (*n* = 77 cells), RPGRIP1L^RNAi^ (*n* = 77 cells), and NPHP4^RNAi^ (*n* = 76 cells) after detergent extraction using 0.01% Triton X-100. Note that in these conditions, more than 80% of cells deciliate. Three replicates per condition. Source data can be found in S3 Data. (E–J) Depletion of CEP290, NPHP4, and RPGRIP1L affects ciliary shape. EM images of abnormal cilia found in NPHP4^RNAi^ (F), RPGRIP1L^RNAi^ (G, H), and CEP290^RNAi^ (I, J). A control cilium is shown in longitudinal and transverse sections in panel A. In NPHP4- and RPGRIP1L-depleted cells, the link between the axoneme and the ciliary membrane is missing at the level of the TZ. Abnormal cilia present extension defects, and vesicles accumulate inside the cilia, probably resulting from a defective TZ gate function. In CEP290-depleted cells, abnormal cilia present a strikingly enlarged lumen, resulting most probably from altered ciliary gate function. Bar: 200 nm. CEP290, centrosomal protein of 290 kDa; EM, electron microscopy; NPHP4, Nephronophtysis 4; polyE, anti-poly-glutamylated tubulin; RNAi, RNA interference; RPGRIP1L, Retinitis pigmentosa GTPase regulator-Interacting Protein 1-Like Protein; TMEM107, Transmembrane protein 107; TZ, transition zone.

Although the depletion of CEP290, RPGRIP1L, and NPHP4 did not modify the ciliary pattern, we wanted to assess whether the deciliation process induced by Ca2+/EtOH buffer might be affected. Ca2+/EtOH induces a massive Ca2+ entry into cells, which activates the molecular machinery that results in the severing of cilia [19]. CEP290^RNAi^, RPGRIP1L^RNAi^, or NPHP4^RNAi^ cells continued to swim after treatment, while control cells immediately stopped moving, suggesting that deciliation was inefficient in these 3 RNAi conditions. Cilia immunostaining confirmed this assumption, since control cells were bald while CEP290-, RPGRIP1L-, or NPHP4-depleted cells remained mostly ciliated (Fig 4A and 4B).

Studies in *Chlamydomonas* showed that the deflagellation process requires both a transient increase of intraciliary Ca2+ and functional microtubule-severing proteins such as katanins and spastins [31,63]. To determine whether the deciliation defects observed after depletion of CEP290 or RPGRIP1L were due to defective Ca2+ signal transduction or to inability of microtubule-severing proteins to break the axoneme, we turned to a dibucaine treatment. This drug is known to increase intracellular and intraciliary calcium concentration by blocking Ca2+ ion extruding channels or by releasing Ca2+ from destabilizing calcium binding proteins and lipids [64]. When exposing RNAi-treated paramecia to 5 mM dibucaine, they rapidly stopped swimming and became bald (Fig 4C). These results indicate that CEP290^RNAi^ or RPGRIP1L^RNAi^ cells possess functional microtubule-severing agents necessary for cilia shedding and that the defects observed can be due to either a defective Ca2+ influx or to the signaling induced by this influx.

To distinguish between these two hypotheses, cells under CEP290^RNAi^, RPGRIP1L^RNAi^, or NPHP4 ^RNAi^ conditions were detergent permeabilized before deciliation. In those permeabilized cells, the Ca2+ present in the deciliation buffer enters the cell and could trigger the transduction signal only if active, resulting in deciliation. Presence or absence of cilia was then determined by IF. Detergent-permeabilized cells deciliate very efficiently using Ca2+/EtOH deciliation buffer (Fig 4D). Altogether, these results suggest that the depletion of CEP290, RPGRIP1L, or NPHP4 leads to defective Ca2+ influx, which may prevent deciliation.

### The conserved domains (coiled-coil region and C2 domains) of RPGRIP1L are required for the deciliation process

In addition to the conserved N-terminal coiled-coil region and central C2 domains [65], *Paramecium* RPGRIP1L displays extra EF-hand domains in the C-terminal part of the protein in place of the RPGR binding domain found in vertebrate RPGRIP1L. The EF-hand domains are known to undergo conformational changes upon calcium binding [66]. We wanted to determine whether cilia autotomy might depend on the conserved coiled-coil and C2 domains or on *Paramecium*-specific C-terminal EF-hand domains. For this, we asked whether a version of RPGRIP1L lacking its EF-hand domains (RPGRIP1LΔEF) but localized like the full-length protein (S6A Fig) could rescue RPGRIP1L depletion. Transformants expressing GFP-tagged RPGRIP1LΔEF were treated with RNAi sequences specifically targeting the endogenous gene (see Schema S6B Fig). As a control, we used transformants expressing the full-length RPGRIP1L-GFP, which is depleted by RNAi treatment and should behave as RPGRIP1L^RNAi^ cells (S6B Fig). Complementation was observed only for transformants expressing GFP-tagged RPGRIP1LΔEF since these cells deciliated like wild-type cells (S6C Fig). Our results confirm that the conserved N-terminal domain is required for the deciliation process and suggest that this function could therefore be conserved in metazoans.

### Depletion of both RPGRIP1L and TMEM107 behaves like the RPGRIP1L^RNAi^

The antagonistic effect of the inactivation of TMEM216/TMEM107 versus RPGRIP1L/NPHP4/CEP290 on the deciliation process led us to study the effect of the double depletion of TMEM107 and RPGRIP1L on this process. The percentage of bald cells observed after Ca2+/EtOH treatment in double inactivated paramecia (TMEM107^RNAi^; RPGRIP1L^RNAi^) is reduced compared to the control (Fig 4B).

To determine whether the hierarchy of incorporation of different proteins in the TZ is compatible with the results of the double depletion of TMEM107 and RPGRIP1L, we examined the fate of fluorescence at the basal bodies in transformants expressing one TZ-GFP protein after knockdown of the other ones (S7 Fig). In keeping with a TZ organizing role, localizations of TMEM107-GFP and TMEM216-GFP are perturbed in CEP290^RNAi^ and clearly diminished in RPGRIP1L^RNAi^ conditions (S7 Fig). These results are in agreement with the epistatic effect of *RPGRIP1L* on *TMEM107* observed in the double knockdown experiment. They are consistent with observations made in *Caenorhabditis elegans*, in which MKS5 (the functional homolog of RPGRIP1L) and CEP290 are required for the localization of MKS module components. An interdependence was observed between TMEM107 and TMEM216 (S7 Fig). Surprisingly, and in contrast with what happens in *C*. *elegans*, CEP290 is required for the proper localization of RPGRIP1L-GFP, but CEP290-GFP localization was not affected by RPGRIP1L depletion.

### Depletion of RPGRIP1L, NPHP4, or CEP290 affects TZ structure

In order to determine whether ultrastructural defects are associated with RPGRIP1L, NPHP4, or CEP290 depletion, we undertook electron microscopy analyses. Even if most of the cilia appeared normal, rare atypical cilia were observed in all types of depleted cells (Fig 4F and 4J). In the case of NPHP4, this is reminiscent of the minor ultrastructural defects observed in mutants of other cell model organisms [3,42]. RPGRIP1L-depleted cells also displayed some aberrant cilia showing developed TZ with clear lack of connections between the TZ region and the membrane (Fig 4G and 4H arrow). Cilia displaying an amazingly enlarged lumen in CEP290-depleted cells (Fig 4I and 4J) were sometimes observed as in *Chlamydomonas* [14] or multiciliated cells of nonsyndromic Leber congenital amaurosis patients harboring *Cep290* mutations [67]. In agreement with IF data, BB anchoring was achieved normally. Altogether, these data suggest that these electron microscopy phenotypes are similar to the ones observed in other cellular models, suggesting that the function of these proteins at the TZ for proper ciliary gating is conserved in *Paramecium*.

## Discussion

The present study of the localization and function of TZ proteins in *Paramecium* is consistent with their known conserved role in ciliary gate structure and function. However, this study also shows—for the first time to our knowledge—that some MKS and NPHP complex proteins, as well as the scaffolding proteins RPGRIP1L and CEP290, have crucial and antagonistic functions in ciliary shedding.

As in other species [40,50,68], the studied *Paramecium* TZ proteins localize with a 9-fold symmetry in several concentric rings located between the tubulin scaffold and the ciliary membrane. All localize along the same axial axis at the level of the axosomal plate, a region closely associated to the ciliary necklace [41]. Such localization is in agreement with the assumption that CEP290, in association with MKS and NPHP modules, organizes the Y-links and the ciliary necklace at the TZ [3,14]. This is reminiscent of the localization on the same axial plane of RPGRIP1L and members of the MKS complex observed in RPE1 primary cilia, whereas, in this case, CEP290 is found below these components [68]. However, it contrasts with the observations performed on cilia/flagella in *Chlamydomonas* [14,42], *Drosophila* [40], and *C*. *elegans* [50], in which CEP290, TMEM107, and TMEM216 extend along the longitudinal length of the TZ. These differences already noticed [9] might reflect the differences in organization of the TZ, which could vary even from one cell type to another in the same organism [69]. In contrast to some other species, only 2 rows constitute the ciliary necklace in *Paramecium*, explaining the localization of the proteins at the same axial level [41].

In the case of RPGRIP1L or NPHP4 gene silencing, the lack of connections between the TZ region and the membrane are clearly observed in some cilia, indicating that these proteins could have a role in tethering the axonemal microtubules to the ciliary membrane. As observed previously in nasal cilia of some nonsyndromic Leber congenital amaurosis patients [67], CEP290-depleted cells display some enlarged cilia, consistent with aberrant gating at the TZ.

By contrast, the depletion of the two MKS complex proteins TMEM216 and TMEM107 does not seem to severely affect the ciliary or the TZ structures. This result is similar to the one observed in other systems such as *C*. *elegans*, in which loss of function of *Tmem107* or *Tmem216* does not lead to gross structural or functional defects as judged by normal dye filling [4,50].

Interestingly, we discovered a novel important function of these proteins in the deciliation process. This was established by the fact that TMEM216- and TMEM107-depleted cells constantly shed their cilia into the extracellular medium by autotomy, followed by ciliary regrowth, whereas RPGRIP1L, CEP290, and NPHP4 by contrast are resistant to deciliation induced by Ca2+/EtOH buffer. Although we cannot exclude a contribution of ciliary involution, our data are consistent with a process of constant deciliation: (i) numerous stretches of tiny or short cilia in the process of growth were observed on the surface of depleted cells (see Fig 2B); (ii) detached cilia were recovered in the culture medium in which depleted cells were grown (graph Fig 2D); and (iii) transcriptomic analysis revealed that one-fourth of the genes differentially regulated after TMEM216 depletion are ciliary genes, half of them also being differentially regulated during the reciliation process (see Fig 3). Together with the results of modulation of ciliary beating experiments (Fig 2E and 2F), these data lead us to propose that the depletion of either TMEM107 or TMEM216 induces a weakening of the distal part of the TZ, which does not resist the shear stress induced by ciliary movements. Interestingly, this finding is consistent with the TZ weakening observed in primary cilia depleted for TCTN2, another TZ protein [70], necessary for the localization of both TMEM216 and TMEM107. The microtubule-severing proteins katanin and spastin have been shown to be involved in ciliary shedding [35,29]. Therefore, we analyzed the expression level of katanin and spastin in our transcriptomic analysis after the depletion of TMEM216/MKS2 or chemical deciliation. No differences were observed in their expression profile in either case, whereas genes encoding proteins involved in membrane fusion—such as *NSF* (an *SNF7* family member) and *VPS4*—were up-regulated. These data reinforce those of Lohret and colleagues [35], who suggested that microtubule-severing proteins are not the primary target of this process but require a Ca2+ dependent activation. By contrast, cilia of RPGRIP1L-, NPHP4-, or CEP290-depleted cells are resistant to Ca2+/EtOH-induced deciliation but shed their cilia either by dibucaine treatment or by detergent permeabilization prior to deciliation. Altogether, these data demonstrate that microtubule severing is not affected in these cells and suggest that the Ca2+ influx, required for deciliation, is deficient in these cells.

Little is known about the deflagellation process that requires both outer microtubule severing and membrane pinch-off at the distal part of the TZ. However, work in *Chlamydomonas* [19] shed some light on this process. Several screens using acidic stress have allowed selection of deflagellation-defective mutants that can be separated into 2 groups [36,39]. The first one consists of mutants supposed to be defective in microtubule-severing events. It includes mutants in the *Fa1* and *Fa2* genes that encode, respectively, a Ca2+/calmodulin-containing protein [37] and a Never in mitosis gene A (NIMA)-related kinase [38]. The second class of mutants (*ADF*) consists of strains that are supposed to be affected in the pathway that activates a calcium influx involved in deflagellation [32,71]: *ADF1* encodes a transient receptor potential (TRP) Ca2+ channel and *ADF3* a conserved microtubule-binding protein, FAP16 [39]. Interestingly, these proteins localized either to the TZ or just above. Since the depletion of CEP290, RPGRIP1L, or NPHP4 disturbs TZ integrity in *Paramecium*, this defect might be accompanied by a mislocalization of proteins involved in the deciliation process such as the TRP calcium channel or FAP16 as described in *Chlamydomonas*. Calcium channel mislocalization is a hypothesis compatible with our results, whereby defective Ca2+ influx would be responsible for the absence of deciliation in CEP290^RNAi^, RPGRIP1L^RNAi^, or NPHP4^RNAi^ cells, as in the *ADF1* mutant of *Chlamydomonas*.

The regulation of phosphoinositide levels at the TZ has also been implicated in flagellar excision. In *Chlamydomonas*, Ca2+ entry during the deflagellation process induces the activation of phospholipase C that triggers the hydrolysis of phosphatidyl inositol 4,5 biphosphate (PIP2) into inositol 1,4,5 triphosphate (I(1,4,5)P3) and Diacylglycerol (DAG) [32,72,73], and the *ADF2* gene might encode an inositol 1,3,4-triphosphate 5,6 kinase, although this is not fully demonstrated [39].

Studies in mammalian cells point to the accumulation of PIP2 in cilia as a “cut here signal,” which leads to cilia decapitation upon mitotic entry [74]. Enriched at the ciliary base in resting cells, the concentration of PIP2 progressively decreases along the TZ and is excluded from the cilia due to its conversion to PIP by an inositol polyphosphate 5-phosphatase (INPP5E), localized in the cilia [75,76]. INPP5E enrichment relies on the integrity of the TZ [77]. The position of this cilia excision signal is correlated with the maximal ciliary PIP2 accumulation. Based on these observations, we propose that a modification of the PIP2 concentration within the TZ could trigger the ciliary autotomy process as already suggested during primary cilium shedding [29]. Inositol lipids are found in *Paramecium* ciliary membranes together with the kinase and phosphatase that interconvert Phosphatidylinositol (PI), Phosphatidylinositol phosphate (PIP), and PIP2 [78]. One can thus suppose that TMEM216 and TMEM107 depletion could affect the distribution of INPP5E, which in turn leads to accumulation of PIP2 and membrane scission. A differential modification of the PIP2 content along the cilia in CEP290-, NPHP4-, or RPGRIP1L-depleted cells compared to the TMEM216- or TMEM107-depleted ones might explain the difference in the resistance to deciliation observed between the different cells.

In metazoans, deciliation has been observed in various mammalian species during the menstrual cycle as, for example, shown in the monkey oviduct [20] and following castration in rabbit [79]. Boisvieux-Ulrich [23], studying deciliation in quail oviduct after progesterone treatment, described two mechanisms responsible for the process. The first is a resorption of the entire axoneme into the cell, and the second is the shedding of cilia into the magnum lumen, which arises by an alteration of the TZ neck region. The latter mechanism occurs on individual or fused cilia, in which the TZ remains individual while the ciliary membrane fuses along the entire length, producing a polyaxoneme within a single membrane. Deciliation in metazoans is not a specific fate of multiciliated cells. Epithelial cells could be reversibly deciliated by diverse agents [80,81]. Recently, an analysis of primary cilium disassembly in IMCD3 by long-term live cell imaging indicated that it occurs predominantly by processes involving ciliary shedding [29]. Finally, apical abscission separating the centrosome from the cilium has been observed during neurogenesis, allowing the detachment of the neurons from the ventricle in order to allow them to migrate to the lateral neural tube [30].

All these results demonstrate that cilia autotomy is a general process occurring in numerous eukaryotes either physiologically or under stress or pharmacological conditions. Our results lead us to propose that, in mammals, the regulation of distal TZ molecular content by MKS/NPHP genes may also be crucial for the cells to trigger cilia autotomy with appropriate timing.

## Materials and methods

### Strains and culture conditions

Stock d4-2 of *P*. *tetraurelia*, the wild-type reference strain, was used in all feeding experiments. The nd7-1 mutant, carrying a recessive monogenic mutation preventing trichocyst discharge [82], was used for the expression of GFP fusions. Cells were grown at 27°C in a wheatgrass infusion (BHB, GSE Vertrieb, Germany), bacterized with *Klebsiella pneumoniae*, and supplemented with 0.8 μg/ml β-sitosterol according to standard procedures [83].

### Gene cloning

Genomic DNA was amplified by PCR, using specific primers (given in S3 Table) in order to fuse the TZ genes to the GFP coding sequence in the pPXV vector, between *Spe*I and *Xho*I restriction sites. If these restriction sites were present in the sequence to be cloned, we used the Gibson cloning method [84]. For gene silencing constructs, sequences of interest were designed for their ability to inactivate all the paralogs, if possible with the help of RNAi off-target tools in ParameciumDB [85] to prevent RNAi off-target. The sequences were cloned into the feeding vector, L4440, between two T7 promoters [86].

## Bioinformatic analyses

Genes were identified using CilDB (http://cildb.i2bc.paris-saclay.fr/) [58,87] and by BLAST search in ParameciumDB (https://paramecium.i2bc.paris-saclay.fr/) [59]. The complete list of genes identified is given in S1 Table. Tophat2 (version 2.0.12 –min-intron-length 15 –max-intron-length 100) was used to map paired-end reads (2 replicates) on the *P*. *tetraurelia* MAC reference (ptetraurelia_mac_51.fa).

Differential gene expression analysis was done with the DESeq2 (version 1.4.1) package. Raw reads were deposited in the ENA under project accession numbers PRJEB33299 and PRJEB33300.

### *Paramecium* transformation

*Nd7-1* cells were transformed by microinjection into their macronucleus [88]. DNA contained a mixture of the plasmid of interest (5 μg/μl) with the DNA directing the expression of the *ND7* gene. Microinjection was made under an inverted Nikon phase-contrast microscope, using a Narishige micromanipulation device and an Eppendorf air pressure microinjector. Transformants were screened for their ability to discharge their trichocysts and further analyzed for GFP.

### Gene silencing

Plasmids used in RNAi silencing experiments were obtained by cloning PCR products about 500 bp, amplified from each gene, in L4440 vector. These plasmids were then transformed in HT115 DE3 *Escherichia coli* strain to produce T7Pol-driven double-stranded RNA as previously described [51]. Paramecia were fed with these bacteria (TZ^*RNAi*^) and refed daily with fresh feeding medium at 27°C. Control cells were fed with HT115 bacteria carrying the L4440 vector containing the *ND7* gene. Phenotypes were analyzed after 48 h of feeding. In these conditions, most of the mRNA of silenced genes are knocked down leading to the depletion of proteins. After the first division upon silencing, parental basal bodies and new basal bodies assembled during the inactivation are mixed within the rows of each daughter cells. Only new basal bodies are affected by the depletion, since TZ components are no longer introduced. In contrast, they are still correctly located in the parental basal bodies.

Efficiency of RNAi was quantified by northern blot for *CEP290*, *RPGRIP1L*, and *NPHP4*. However, it was not possible to perform this experiment for *TMEM107* and *TMEM216* because the double-stranded RNA used for silencing covered the targeted genes in their entirety. This is because, in *Paramecium*, RNAi is done by feeding the cells with bacteria producing long (>500 bp) double-stranded RNA complementary to all or part of the target mRNA. The double-stranded RNA produces numerous different 23 nt siRNA [52]. In the case of *TMEM107* and *TMEM216*, the produced double-stranded RNA corresponds to the entire gene. This prevents the test of efficiency by northern blot. In these cases, we quantified the decrease of BB fluorescence in transformed cell lines expressing the GFP-tagged transgene after silencing.

In *Paramecium*, the phenotype of silenced cells is highly reproducible from one experiment to another and from one cell to another. In each experiment, more than 50 cells are analyzed in at least 2 or 3 independent experiments.

### Paramecia fixation for IF

Fixation and IF techniques are performed on cells in suspension. The amount of 50–100 cells were collected in the smallest volume possible and were permeabilized in 200 μl PHEM (Pipes 60 mM, Hepes 25 mM, EGTA 10 mM, MgCl_2_ 2 mM, adjusted to pH 6.9) with 0.5% saponin (PHEM-saponin) or with 0.5% Triton-X100 (PHEM-Triton) for 30 s. After using saponin for cell permeabilization, 0.5% of saponin was added to each buffer afterwards. Cells were fixed for 15 min after adding an equal volume of PHEM, 2% PFA. Buffer was then aspirated, and cells were rinsed 3 times for 10 min in PHEM, 0.5% saponin.

In order to ascertain that our immunostaining protocol was not affecting the ciliation pattern, ink-labeled controls were always mixed with RNAi-treated cells and all cells treated simultaneously within the same dish. India or red ink was added to control cultures at least 30 min before staining. India ink or red ink particles are nontoxic for the cells but are absorbed in the feeding vacuoles, which allows detection of control cells by their black or red vacuoles using phase contrast. The unlabeled cells are referred to TZ^RNAi^.

Cilia are often more fragile when paramecia are taken directly from 27°C, the usual growth temperature, so cells had to be kept 1 or 2 h at room temperature before proceeding to fixation.

### IF

All antibodies, diluted in PHEM, 0.5% saponin, were incubated for 15 min. After 3 washes, cells were incubated with the appropriate secondary antibodies from Thermofisher at a dilution of 1:500 together with DAPI. Two final washes were done, and cells were mounted in citifluor AF1 (EMS, Hatfield, PA). The following antibodies were used: the monoclonal anti-tubulin ID5 (1:100; recognizes poly-glutamylation of α-tubulin [89,90] [a gift from J. Wehland], stained the basal bodies, and slightly stained the cilia); the monoclonal TAP952 (which stained the tip of cilia) and AXO49 (from Callen and colleagues [91], which labeled the cilia [culture supernatant 1/50]), recognize, respectively, the monoglycylated and the polyglycylated tubulins [92]; the polyclonal PolyE (1:100) anti-tubulin [93] (which stained cilia and was provided generously by C. Janke); and polyclonal anti-GFP antibodies (1:1000, Interchim, Montlucon, France; stained GFP-flagged proteins). For more details on IF methods and antibody specificity, see Aubusson-Fleury and colleagues [92].

Confocal acquisitions were made with a Leica SP8 equipped with a UV diode (line 405), 3 laser diodes (lines 488, 552 and 635) for excitation, and 2 PMT detectors. Image stacks were processed with ImageJ and Photoshop. Confocal acquisition parameters were kept identical (same laser intensity, same gain, and same resolution) for quantification of BB fluorescence intensity in control and RNAi^TZ^-treated cells. Average pixel fluorescence intensity of GFP at the BB was then measured within a defined and constant circular area of the size of the BB, using the measurement tools of Image J. Quantification data were compiled from measurements of 100 BB on 5 paramecia from 2 different experiments.

### STED analysis

STED imaging was performed using a Leica TCS SP8 STED 3X (Leica Microsystems CMS GmbH, Mannheim, Germany). The system was equipped with a WLL ranging from 470 to 670 nm for excitation and with three-dimensional STED lasers at 592, 660, and 775 nm. A 100× 1.4 Oil STED white objective was used to acquire the images. GFP, AF568, and Cy5 were excited at 488, 561, and 643 nm, respectively. Detection ranges were 500–550, 575–625, and 660–700 nm, respectively. A pixel size of 25 nm was used. For deconvolution, SVI Huygens was used. Diameters were measured using the plot profile tool in ImageJ to obtain the distance between the 2 maximum intensity peaks.

### Electron microscopy

For ultrastructural observations, cells were fixed in 1% (v/v) glutaraldehyde and 1% OsO4 (v/v) in 0.05 M cacodylate buffer (pH 7.4) for 30 min. After rinsing, cells were embedded in 2% agarose. Agarose blocks were then dehydrated in graded series of ethanol and propylene oxide and embedded in Epon812 (TAAB, Aldermaston, Berkshire, UK). For pre-embedding immunolocalization, the immunostaining process was carried out as described for IF using gold-coupled instead of fluorochrome-coupled secondary antibodies (gold-labeled anti-rabbit IgG-GAR G10, Aurion) diluted 1/50 for 30 min. Cells were then treated as described earlier.

All ultrathin sections were contrasted with uranyl acetate and lead citrate. The sections were examined with a Jeol transmission electron microscope 1,400 (at 120 kV).

### Swimming analysis

Four to eight paramecia were transferred in 10 μl drops of conditioned BHB (bacterized BHB solution, then depleted of bacteria after their growth and sterilized) for 15 min before being tracked for 10 s every 0.3 s. We used a Zeiss Steni 2000-C dissecting microscope with 1-min time-lapse acquisitions at 7 frames per second with a Roper Coolsnap-CF camera and Metamorph software (Universal Imaging). Stacks were analyzed using the Manual tracking tool in ImageJ.

### Quantification of free cilia in culture medium

After 24 h of gene silencing, cells were transferred into fresh medium in order to obtain 10 cells per 300 μl well at t = 48 h. The number of cells was adjusted according to the dividing capacity of the RNAi condition. At 48 h of RNAi treatment, all the cells were carefully aspirated from the culture wells in order to collect the amount of medium corresponding to 300 cells (approximately 30–35 wells per condition). The culture medium was then centrifuged onto a microscopy coverslip as previously described for centrosomes [94]. Cilia were fixed with Methanol-20°C for 6 min and stained using TAP952 and polyE antibodies. The number of cilia per microscope field (184.7 × 184.7 μm) was quantified (10 fields).

### Deciliation experiments

#### Standard protocol

Paramecia (RNAi-treated and ink-labelled controls) were transferred to 1 mL Ca2+/EtOH deciliation buffer (10 mM Tris, 1 mM Ca2+, 5% EtOH) and vigorously vortexed for 2 min to allow the cilia to shed.

#### Dibucaine treatment

Cells were resuspended in a solution containing 5 mM MgSO4, 4% sucrose, and 25 mM Hepes. Dibucaine-HCl (Sigma) 25 mM was added to obtain a final concentration of 5 mM according to Nelson [95].

#### TritonX-100 treatment

Cells were first permeabilized with 0.01% Triton in DRYL buffer (2 mM sodium citrate, 1 mM NaH2PO4, 1 mM Na2HPO4, 1.5 mM CaCl2) [96] for 1 min and then transferred in the deciliation buffer and treated as in the standard protocol.

#### PEG treatment

Cells were grown for 1 h in 10% PEG6000 (Merck) (diluted in conditioned BHB).

After deciliation treatment, cells were rinsed and immunostained for cilia and observed under the epifluorescence microscope. Cells were classified into 4 categories according to their ciliation state: normal, 75% cilia (= few holes in the ciliation), 50% cilia, and bald (less than 25% of cilia).

### Northern blot analysis

Northern blots were carried out as follows: total RNA samples (20 μg) were denatured in Glyoxal load Dye (Ambion) for 30 min at 50°C before loading on 1% agarose gels for electrophoresis. Gels were transferred to Hybond N+membrane in 20XSSC buffer and UV cross-linked. Hybridization was carried out overnight in 7% SDS, 0.5 M sodium phosphate, 1% BSA, and 1 mM EDTA (pH 7.4) at 65°C or 50°C. Double-stranded probes were labelled by random priming with [α-32P] dATP (3000 Ci/mmol, PerkinElemer) and DNA polymerase I Large (Klenow) fragment (Promega). The 17S rRNA oligonucleotide probe, used as loading control, was labelled with [γ-32P] ATP (5,000 Ci/mmol; HARTMANN ANALYTIC) and T4 polynucleotide kinase (Biolabs). Membranes were then washed successively in 20XSSC, 0.1% SDS and 0.2XSSC, 0.1% SDS for 30 min at 60°C or 50°C prior to exposure on storage phosphor screen and scan on Typhoon biomolecule imager (GE Healthcare).

### Statistical analyses

For determining ciliated versus bald cells, we obtained paired observations on 2 variables for which we wanted to assess the independence of 2 populations. In those cases, the events being considered were mutually exclusive, and the total probability was 1. All observations were independent. Therefore, we used the χ^2^ test. All calculations were made with GraphPad Prism 6. All error bars show SD.

For BB fluorescence quantification and swimming velocity experiment, statistical significance was assessed using a *t* test. All calculations were made with GraphPad Prism 6. All error bars show SD.

## Supporting information

S1 Fig *Paramecium* TZ proteins are recruited when cilia extend. (A) Localization of TZ protein–GFP fusions in paramecia fixed before permeabilization. Labelling using both 2 monoclonal antibodies ID5 (decorating BB and cilia in magenta) and Axo49 (decorating cilia, also in magenta) and a polyclonal anti-GFP (in green). TMEM216-GFP localizes both at the TZ of ciliated basal bodies and at the proximal side of nonciliated ones. This proximal signal completely disappears in cells permeabilized before fixation (see Fig 2A). Bar = 1 μm. (B) Cilia at different steps of their growth are observed in dividing transformed TZ-GFP cells, which harbor numerous growing cilia. Cells are stained using GFP and ID5 antibodies. To avoid fluctuations in fluorescence intensity due to variations in expression level, all the cilia were taken within a same cell for each expressing TZ-GFP as indicated. Cilia were classified according to their length, and the presence of GFP signal at the TZ was observed. The GFP signal is detected at the TZ as soon as the growing cilium is detected by ID5 antibodies.
(TIF)

S2 Fig Efficiency of inactivation of the different RNAi vectors. (A) Northern blot analysis (left) of expression levels of CEP290, NPHP4, and RPGRIP1L genes in ND7^RNAi^ (Control) and CEP290^RNAi^, NPHP4^RNAi^, and RPGRIP1L^RNAi^. Signals were quantified and normalized with the 17S rRNA signal used as loading control. CEP290 and RPGRIP1L probes target the mRNAs of the 2 paralogs of each gene, since the genes are nearly identical. Three different probes (noted NPHP4 sc2, NPHP4 sc13, and NPHP4 sc29) were used for NPHP4 since paralogs are divergent. Right panel: histogram showing the decrease of each mRNA compared to the control. For each gene family, RNAi triggers a decrease of at least 40% of mRNA. Source data can be found in S4 Data. (B) Quantification of the GFP fluorescence remaining at the BB after 24 h of TMEM107^RNAi^ observed in TMEM107 GFP transformants compared to the control RNAi. BB counted: 100 on 5 paramecia from 2 different experiments. Unpaired two-sided *t* test, ****p < 0.0001. Source data can be found in S4 Data. (C) Quantification of the GFP fluorescence remaining at the BB after 24 h of TMEM216^RNAi^ observed in TMEM216 GFP transformants compared to the control RNAi. BB counted: 100 on 5 paramecia from 2 different experiments. Unpaired two-sided *t* test ****p < 0.0001. Source data can be found in S4 Data. a.u., arbitrary units.
(TIF)

S3 Fig Cell division number and swimming velocity after TZ protein depletion. (A) Curves depicting the cell division number observed after 24 h, 48 h, and 72 h of TZ^RNAi^ compared to controlRNAi. Source data can be found in S5 Data. (B) Dot plot graph depicting the mean swimming speeds of control paramecia and depleted cells after 24 h and 48 h of feeding. Each dot shows the mean velocity of 1 cell (*n* ≥ 120 cells per condition performed in 3 independent replicates). Mean velocity after 48 h of depletion: Control 770 μm/s, TMEM107RNAi 341 μm/s, TMEM216 RNAi 307 μm/s, CEP290 RNAi 319 μm/s, RPGRIP1L RNAi 385 μm/s, NPHP4 RNAi 493 μm/s. The lines represent the mean and the error bars the standard deviation. Statistical significance was assessed by unpaired two-sided *t* test, ****p < 0.0001. Source data can be found in S5 Data.
(TIF)

S4 Fig Depletion of TZ proteins does not affect BB positioning. Paramecia were decorated for basal bodies and cilia using the polyclonal poly-glutamylated tubulin (poly-E) antibodies. Basal bodies are perfectly aligned along ciliary rows indicating an absence of BB duplication or anchoring defects. Bar = 15 μm.
(TIF)

S5 Fig TMEM107- and TMEM216-depleted cells shed their cilia distally of the TZ. Other examples of basal bodies harboring an extended TZ specific of ciliated ones and severed above the axosomal plate, observed after the depletion of either TMEM107 or TMEM216. The TZ is indicated by a red arrow. This indicates that the cilia have been shed. Bar = 200 nm.
(TIF)

S6 Fig RPGRIP1L EF-hand domains are not involved in the deciliation signal. (A) Localization of RPGRIP1L-GFP full-length (FL; left) and RPGRIP1L short form-GFP (RPGRIP1LΔEFhands). These two proteins localize similarly. Bar = 10 μm. (B) Experimental design: paramecia cell lines expressing transgenes encoding either the RPGRIP1L-GFP full-length or the RPGRIP1LΔEF-hands-GFP were generated. The 2 different transformed cell lines were then inactivated by RNAi sequences specifically targeting the endogenous gene (EF-hand domain). As a control, expressed RPGRIP1L-GFP full-length RNAi degradable was used while RPGRIP1LΔEF-hands-GFP resistant to RNAi might complement the depletion of RPGRIP1L. The black cross on the protein schemas indicate that the protein will not be produced due to the RNAi. (C) Bar plot showing the quantification of ciliated cells observed after Ca2+/EtOH treatment of RPGRIP1L-FL expressing cells (FL) or RPGRIP1LΔEF expressing cells (Δ) after silencing (controlRNAi or EF-hand domain^RNAi^). Source data can be found in S6 Data. For FL, number of analyzed cells: Control^RNAi^ (*n* = 126 cells), EF-Hand^RNAi^ (*n* = 94 cells). For ΔΔ number of analyzed cells: Control^RNAi^ (*n* = 90 cells), EF-Hand^RNAi^ (*n* = 158 cells). Error bars represent the standard deviation. >2 independent replicates per condition. Statistical significance was assessed by unpaired two-sided χ^2^ test, ****p < 0.0001. Source data can be found in S6 Data.
(TIF)

S7 Fig Hierarchy of incorporation of TZ proteins. (A) TMEM107-GFP transformants were treated with Control^RNAi^, TMEM216^RNAi^, RPGRIP1L^RNAi^, and CEP290^RNAi^. Control^RNAi^ and TMEM107^RNAi^ were used to quantify the efficiency of the silencing (see S2B Fig). Note that either CEP290^RNAi^, RPGRIP1L^RNAi^, or TMEM 216^RNAi^ prevent the localization of TMEM107-GFP at the BB. (B) TMEM216-GFP transformants were treated with Control^RNAi^, TMEM107^RNAi^, RPGRIP1L^RNAi^, and CEP290^RNAi^. Control^RNAi^ and TMEM216^RNAi^ were used to quantify the efficiency of the silencing (see S2B Fig). Note that either CEP290^RNAi^, RPGRIP1L^RNAi^, or TMEM 216^RNAi^ prevent the localization of TMEM216-GFP at BB. (C) CEP290-GFP transformants were treated with Control^RNAi^, TMEM107^RNAi^, TMEM216^RNAi^, and RPGRIP1L^RNAi^. CEP290^RNAi^ is used as a control. Note that the depletion of TMEM107, TMEM216, and RPGRIP1L do not modify the CEP290-GFP pattern, while depletion of CEP290 is greatly diminished, which demonstrates the silencing efficiency. (D) RPGRIP1L-GFP transformants were treated with Control^RNAi^, TMEM107^RNAi^, TMEM216^RNAi^, and CEP290^RNAi^. RPGRIP1L^RNAi^ is used as a control. Note that only CEP290^RNAi^ prevents the localization of RPGRIP1L-GFP at the BB, while the depletion of TMEM107 and TMEM216 do not modify the RPGRIP1L-GFP pattern. See also the disappearance of the fluorescence after RPGRIP1L^RNAi^, which shows the silencing efficiency. Left panels: entire cell; right panels: magnification of BB rows. Bar: 10 μm.
(TIF)

S1 Table TZ proteins identified in *Paramecium*. ID of TZ proteins in *Paramecium*.
(PDF)

S2 Table List of genes differentially expressed in silenced TMEM216 cells. Sheet 1: *Paramecium* genes whose expression was significantly modified in TMEM216^RNAi^ cells. The genes mentioned in the text are highlighted in yellow. Sheet 2: *Paramecium* genes whose expression is significantly modified both in TMEM216^RNAi^ cells and during reciliation. Sheet 3: Potential ciliary genes identified by proteomic and comparative genomic analyses whose expression is significantly modified in TMEM216^RNAi^ cells. Sheet 4: *Paramecium* genes, not identified as ciliary genes, whose expression is significantly modified in TMEM216^RNAi^ cells. ID: ParameciumDB accession number. FC_TMEM216: Fold change value compared to the control. NB_ciliary_evidence: number of high-throughput ciliary studies in which the gene or its homologs were identified as a potential ciliary gene. NB_PEP_Yano: number of peptides assigned to the encoded *protein* identified in the proteome of the ciliary membrane in *Paramecium* [61] NA: no peptide identified. Ciliary proteome: peptides assigned to the protein encoded by the gene were identified in the *Paramecium* ciliary proteome [58]. Reciliation_transcriptome_significant: expression of the gene was significantly modified during the reciliation process [59]. Reciliation_transcriptome_cluster_att. The genes differentially expressed during reciliation were clustered according to their expression profile [59]. This column indicates the cluster to which the gene was assigned. No cluster indicates that its expression was not significantly modified. ANNOTATION: predicted function of the protein interpro_desc_terms: InterPro predicted function of the protein.
(XLSX)

S3 Table Primer sequences. Primer sequences used in this study.
(XLSX)

S1 Data Mean TZ length of ciliated and unciliated BB, including raw data from individual TZ and mean TZ diameter corresponding to the 5 studied proteins as well as tubulin, including raw data. Data corresponding with Fig 1C and Fig 1F.
(XLSX)

S2 Data Mean percentage of short cilia and free cilia in the supernatant after normalization, as well as the effect of beating forces on ciliary shedding. All raw data are included. Data corresponding with Fig 2C–2F.
(XLSX)

S3 Data Mean percentage of bald cells after deciliation with Ca2+/EtOH or dibucaine, or after permeabilization with Triton X-100. All raw data are included. Data corresponding with Fig 4C and 4D.
(XLSX)

S4 Data Quantification of mRNA levels and BB fluorescence intensity after RNAi silencing. For the blots shown in S2 Fig, the histogram shows the signals measured for each mRNA gene after normalization with the signals from the 17S rRNA probe, relatively to the signal obtained in the Nd7 control silencing. All raw data are included. Data corresponding with S2A, S2B and S2C Fig.
(XLSX)

S5 Data Growth and swimming velocity after RNAi depletion. All raw data are included. Data corresponding with S3A and S3B Fig.
(XLSX)

S6 Data Quantification of ciliated cells observed after Ca2+/EtOH treatment of RPGRIP1L-FL expressing cells (FL) or RPGRIP1LΔEF expressing cells (Δ) after silencing (control RNAi or EF-hand domain RNAi). All raw data are included. Data corresponding with S6C Fig.
(XLSX)

## Abbreviations

BB: basal body
Bug22: Basal body up-regulated protein 22
CEP290: Centrosomal protein of 290 kDa
DAG: Diacylglycerol
DNAH2: dynein axonemal heavy chain 2
Fa1p: Flagellar automy 1p
Fa2p: Flagellar automy 2p
FAP16: Flagellar associated protein 16
GFP: green fluorescent protein
I(1,4,5)P3: inositol 1,4,5 triphosphate
IF: immunofluorescence
IFT57: intraflagellar transport 57
IMCD3: inner medullary collecting duct 3
INPP5E: inositol polyphosphate 5-phosphatase
MKS: Meckel Gruber syndrome
NEK: Never in mitosis A related kinase
NIMA: Never in mitosis gene A
NPHP: Nephronophtysis
PEG: Polyethylene glycol
PI: Phosphatidylinositol
PIP: Phosphatidylinositol phosphate
PIP2: phosphatidyl inositol 4,5 biphosphate
RNAi: RNA interference
RPGR: Retinitis pigmentosa GTPase regulator
RPGRIP1L: RPGR-Interacting Protein 1-Like Protein
STED: Stimulated Emission Depletion
TEM: transmission electron microscopy
TMEM107: Transmembrane protein 107
TRP: transient receptor potential
TZ: transition zone
