# Supplementary figures and images for: MKS-NPHP module proteins control ciliary shedding at the transition zone
Source: PLoS Biol. 2020 Mar 12;18(3):e3000640. doi: 10.1371/journal.pbio.3000640 (PMC7093003; doi:10.1371/journal.pbio.3000640)

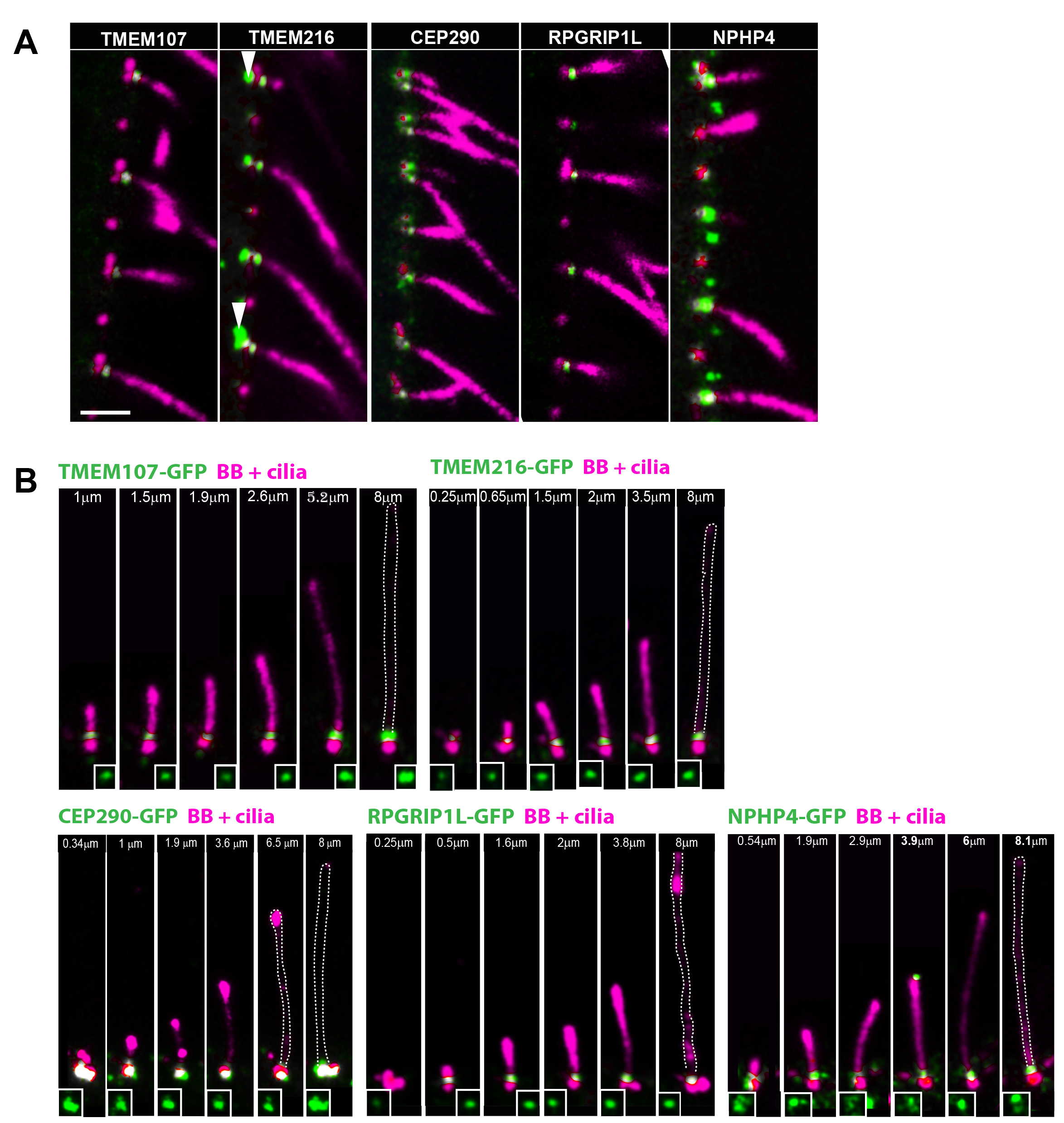

Supplement: S1 Fig — (A) Localization of TZ protein–GFP fusions in paramecia fixed before permeabilization. Labelling using both 2 monoclonal antibodies ID5 (decorating BB and cilia in magenta) and Axo49 (decorating cilia, also in magenta) and a polyclonal anti-GFP (in green). TMEM216-GFP localizes both at the TZ of ciliated basal bodies and at the proximal side of nonciliated ones. This proximal signal completely disappears in cells permeabilized before fixation (see Fig 2A). Bar = 1 μm. (B) Cilia at different steps of their growth are observed in dividing transformed TZ-GFP cells, which harbor numerous growing cilia. Cells are stained using GFP and ID5 antibodies. To avoid fluctuations in fluorescence intensity due to variations in expression level, all the cilia were taken within a same cell for each expressing TZ-GFP as indicated. Cilia were classified according to their length, and the presence of GFP signal at the TZ was observed. The GFP signal is detected at the TZ as soon as the growing cilium is detected by ID5 antibodies. (TIF) [file pbio.3000640.s001.tif]

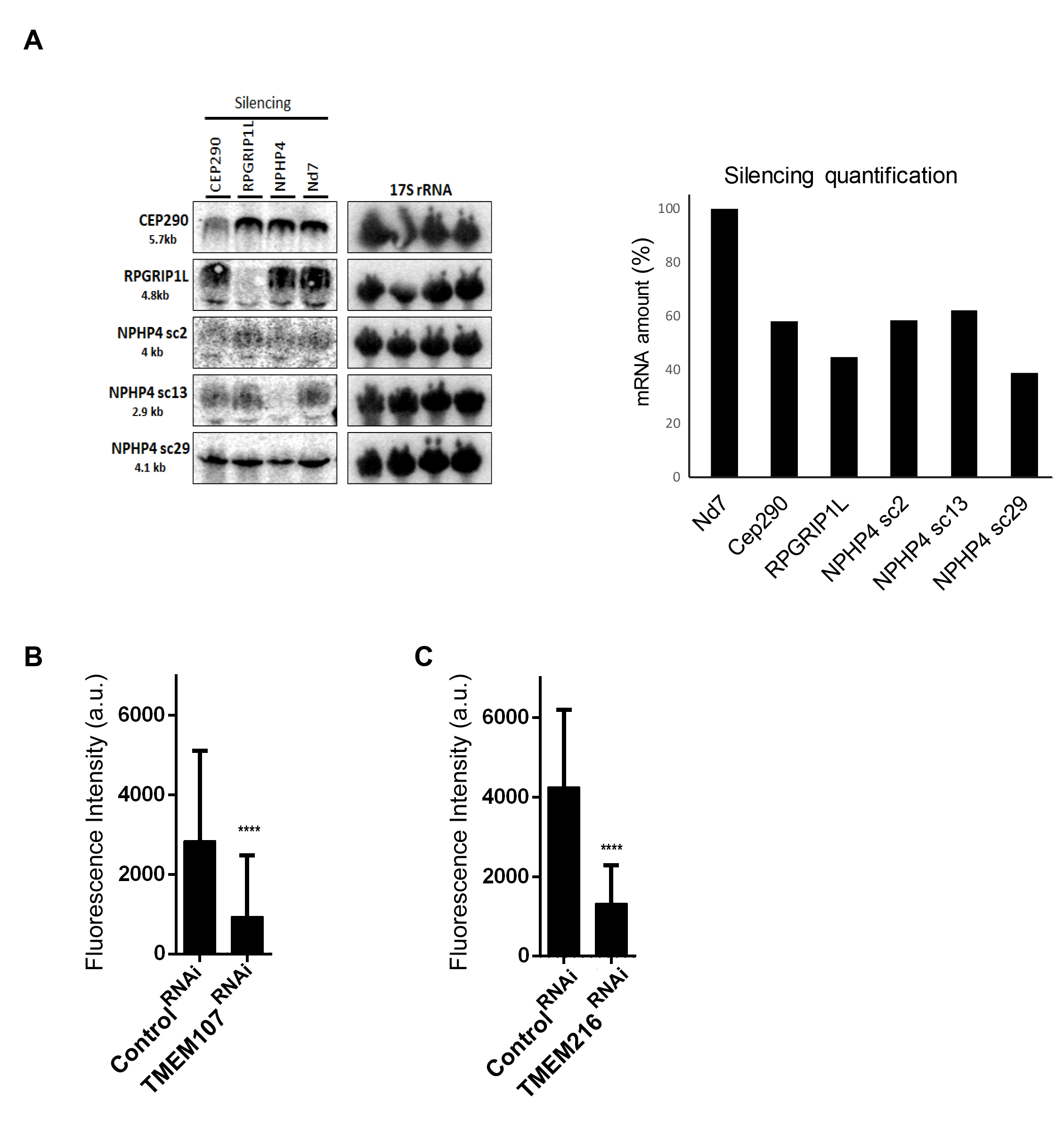

Supplement: S2 Fig — (A) Northern blot analysis (left) of expression levels of CEP290, NPHP4, and RPGRIP1L genes in ND7RNAi (Control) and CEP290RNAi, NPHP4RNAi, and RPGRIP1LRNAi. Signals were quantified and normalized with the 17S rRNA signal used as loading control. CEP290 and RPGRIP1L probes target the mRNAs of the 2 paralogs of each gene, since the genes are nearly identical. Three different probes (noted NPHP4 sc2, NPHP4 sc13, and NPHP4 sc29) were used for NPHP4 since paralogs are divergent. Right panel: histogram showing the decrease of each mRNA compared to the control. For each gene family, RNAi triggers a decrease of at least 40% of mRNA. Source data can be found in S4 Data. (B) Quantification of the GFP fluorescence remaining at the BB after 24 h of TMEM107RNAi observed in TMEM107 GFP transformants compared to the control RNAi. BB counted: 100 on 5 paramecia from 2 different experiments. Unpaired two-sided t test, ****p < 0.0001. Source data can be found in S4 Data. (C) Quantification of the GFP fluorescence remaining at the BB after 24 h of TMEM216RNAi observed in TMEM216 GFP transformants compared to the control RNAi. BB counted: 100 on 5 paramecia from 2 different experiments. Unpaired two-sided t test ****p < 0.0001. Source data can be found in S4 Data. a.u., arbitrary units. (TIF) [file pbio.3000640.s002.tif]

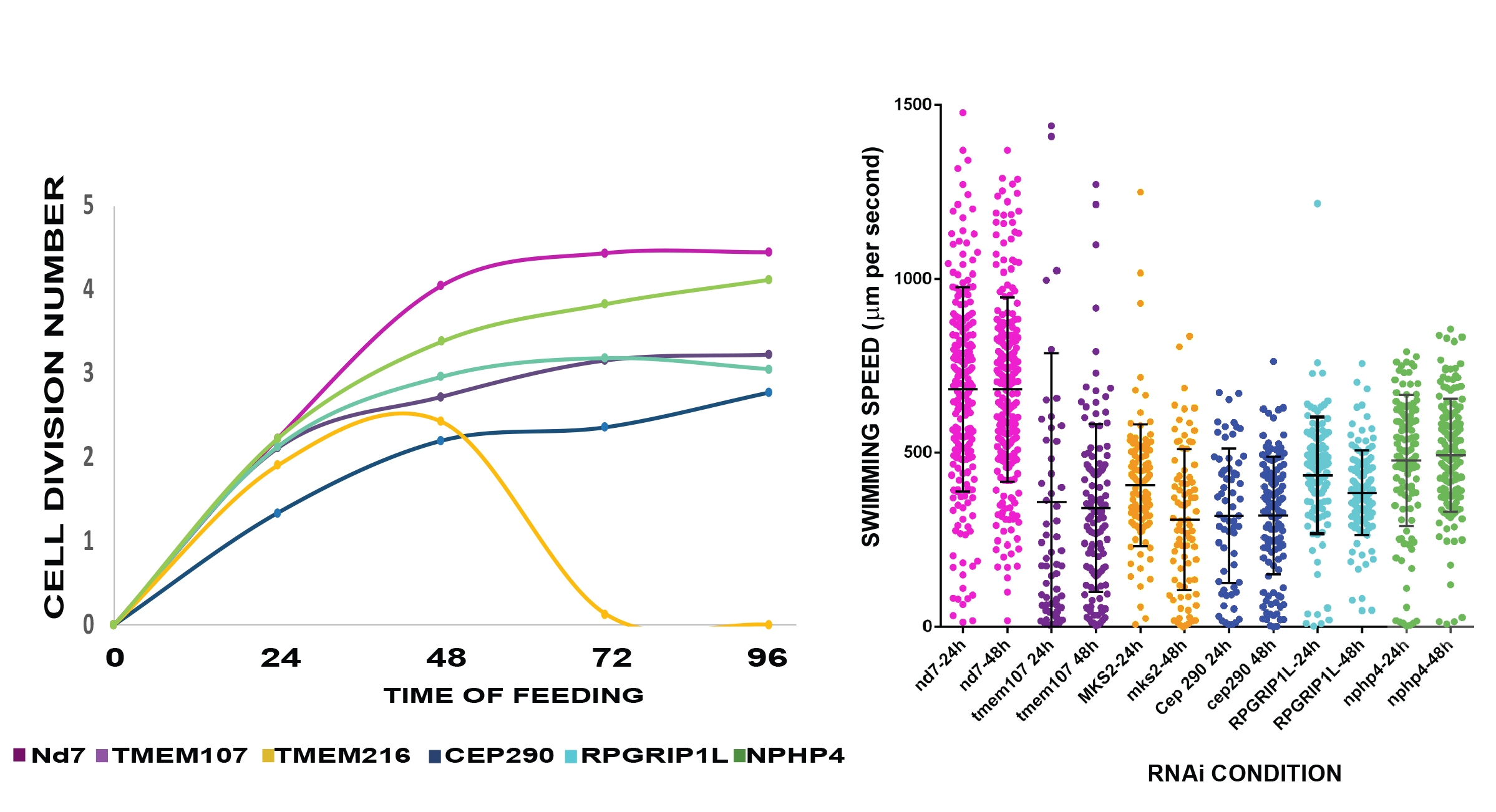

Supplement: S3 Fig — (A) Curves depicting the cell division number observed after 24 h, 48 h, and 72 h of TZRNAi compared to controlRNAi. Source data can be found in S5 Data. (B) Dot plot graph depicting the mean swimming speeds of control paramecia and depleted cells after 24 h and 48 h of feeding. Each dot shows the mean velocity of 1 cell (n ≥ 120 cells per condition performed in 3 independent replicates). Mean velocity after 48 h of depletion: Control 770 μm/s, TMEM107RNAi 341 μm/s, TMEM216 RNAi 307 μm/s, CEP290 RNAi 319 μm/s, RPGRIP1L RNAi 385 μm/s, NPHP4 RNAi 493 μm/s. The lines represent the mean and the error bars the standard deviation. Statistical significance was assessed by unpaired two-sided t test, ****p < 0.0001. Source data can be found in S5 Data. (TIF) [file pbio.3000640.s003.tif]

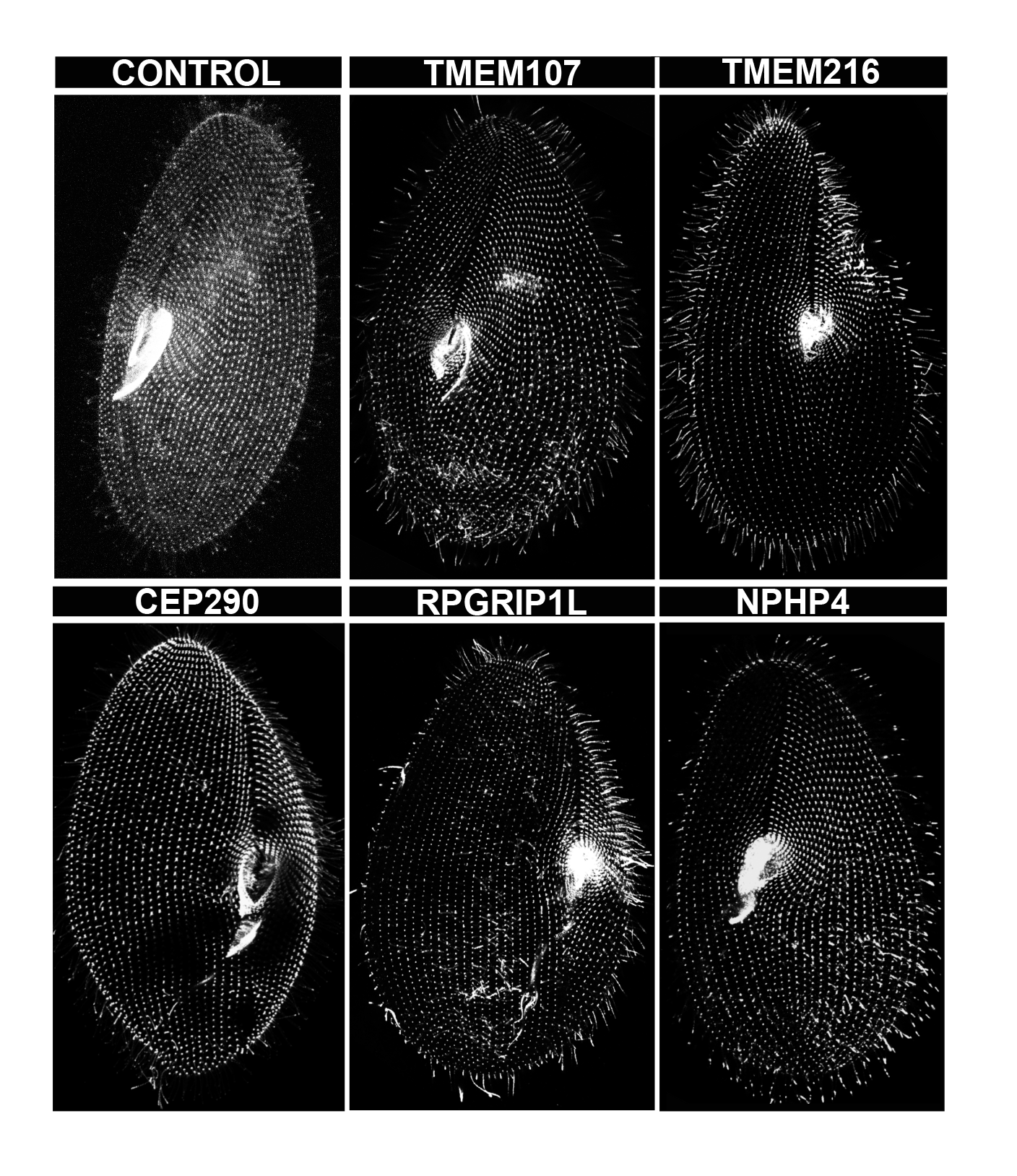

Supplement: S4 Fig — Paramecia were decorated for basal bodies and cilia using the polyclonal poly-glutamylated tubulin (poly-E) antibodies. Basal bodies are perfectly aligned along ciliary rows indicating an absence of BB duplication or anchoring defects. Bar = 15 μm. (TIF) [file pbio.3000640.s004.tif]

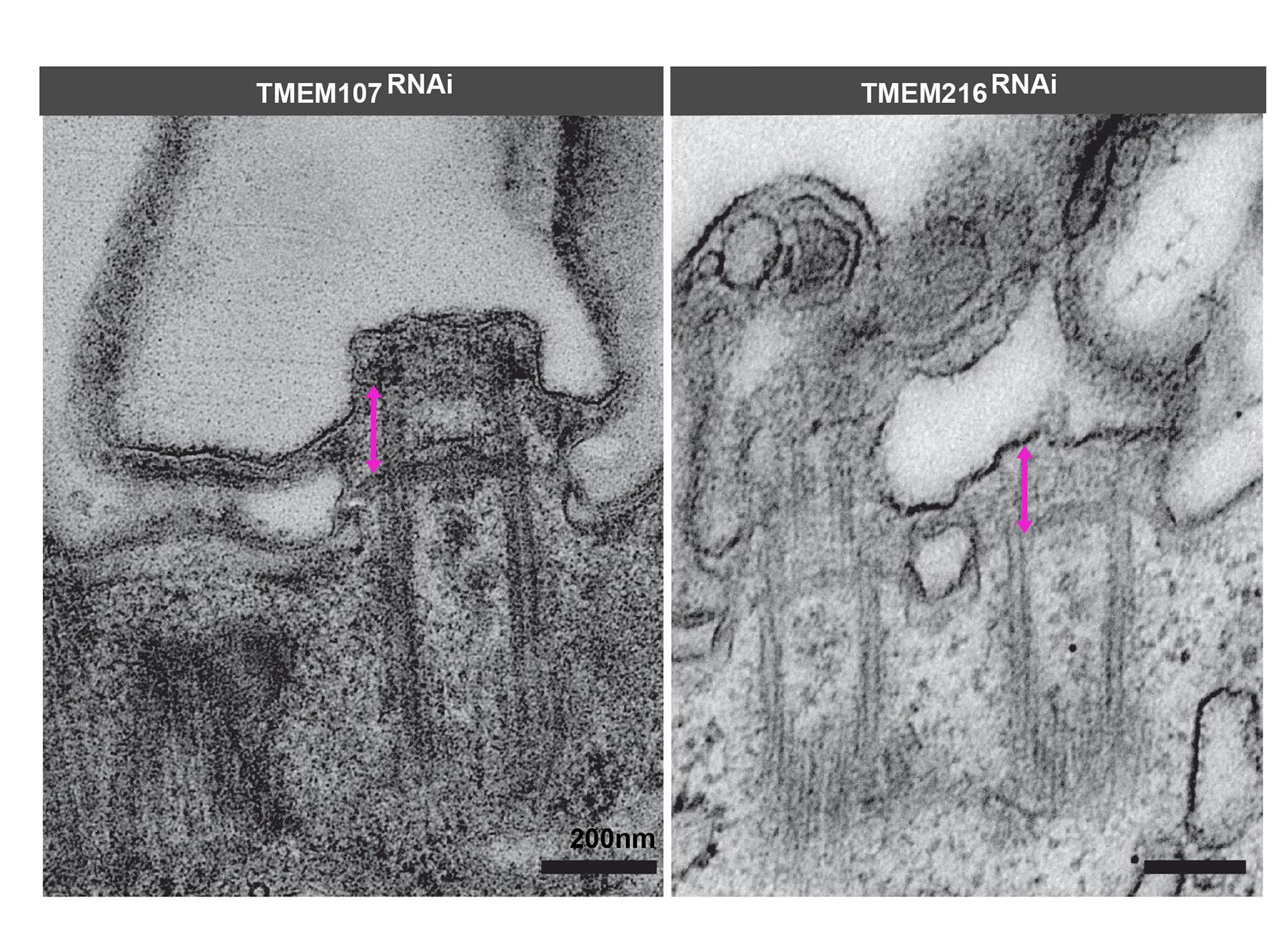

Supplement: S5 Fig — Other examples of basal bodies harboring an extended TZ specific of ciliated ones and severed above the axosomal plate, observed after the depletion of either TMEM107 or TMEM216. The TZ is indicated by a red arrow. This indicates that the cilia have been shed. Bar = 200 nm. (TIF) [file pbio.3000640.s005.tif]

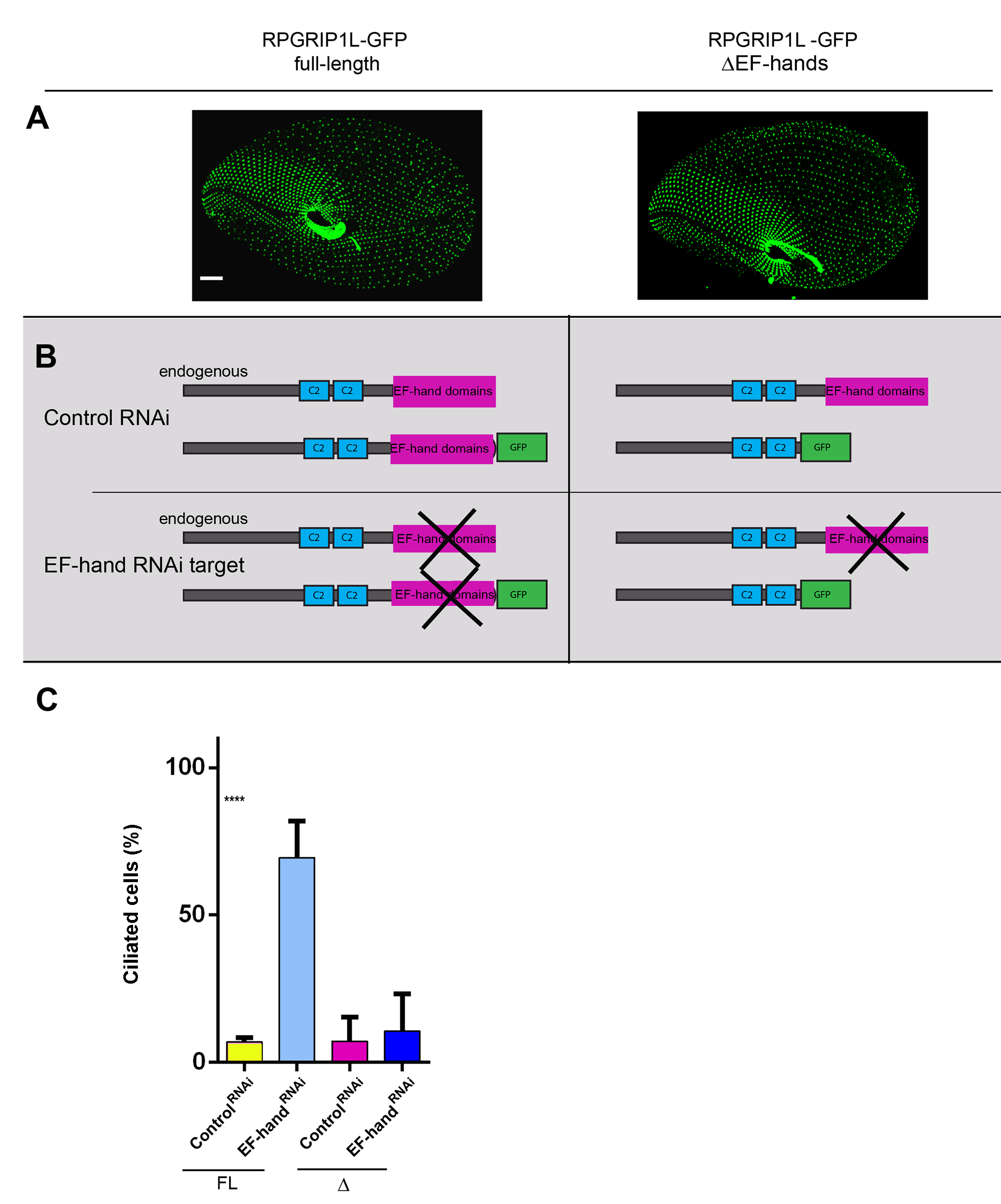

Supplement: S6 Fig — (A) Localization of RPGRIP1L-GFP full-length (FL; left) and RPGRIP1L short form-GFP (RPGRIP1LΔEFhands). These two proteins localize similarly. Bar = 10 μm. (B) Experimental design: paramecia cell lines expressing transgenes encoding either the RPGRIP1L-GFP full-length or the RPGRIP1LΔEF-hands-GFP were generated. The 2 different transformed cell lines were then inactivated by RNAi sequences specifically targeting the endogenous gene (EF-hand domain). As a control, expressed RPGRIP1L-GFP full-length RNAi degradable was used while RPGRIP1LΔEF-hands-GFP resistant to RNAi might complement the depletion of RPGRIP1L. The black cross on the protein schemas indicate that the protein will not be produced due to the RNAi. (C) Bar plot showing the quantification of ciliated cells observed after Ca2+/EtOH treatment of RPGRIP1L-FL expressing cells (FL) or RPGRIP1LΔEF expressing cells (Δ) after silencing (controlRNAi or EF-hand domainRNAi). Source data can be found in S6 Data. For FL, number of analyzed cells: ControlRNAi (n = 126 cells), EF-HandRNAi (n = 94 cells). For ΔΔ number of analyzed cells: ControlRNAi (n = 90 cells), EF-HandRNAi (n = 158 cells). Error bars represent the standard deviation. >2 independent replicates per condition. Statistical significance was assessed by unpaired two-sided χ2 test, ****p < 0.0001. Source data can be found in S6 Data. (TIF) [file pbio.3000640.s006.tif]

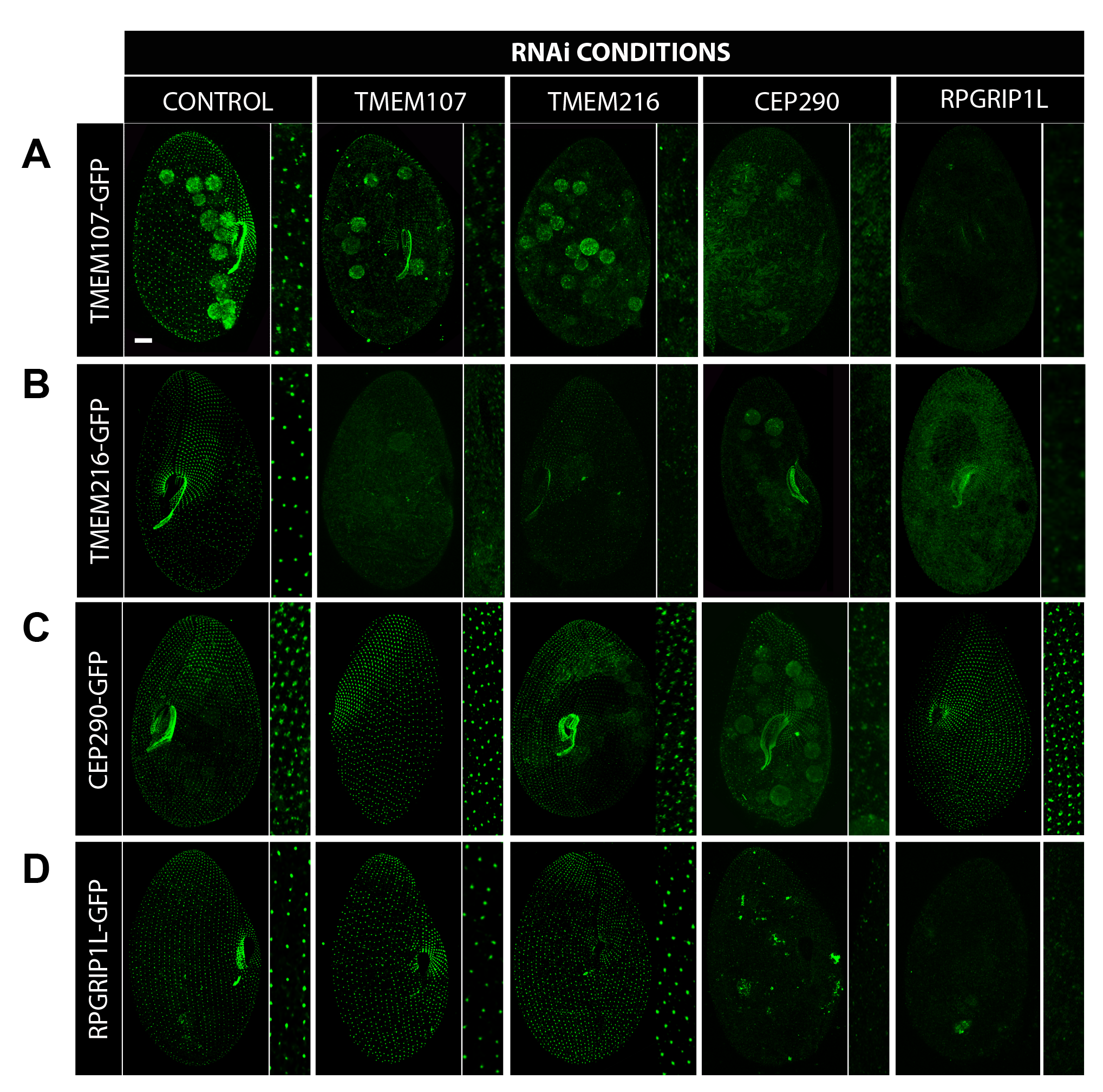

Supplement: S7 Fig — (A) TMEM107-GFP transformants were treated with ControlRNAi, TMEM216RNAi, RPGRIP1LRNAi, and CEP290RNAi. ControlRNAi and TMEM107RNAi were used to quantify the efficiency of the silencing (see S2B Fig). Note that either CEP290RNAi, RPGRIP1LRNAi, or TMEM 216RNAi prevent the localization of TMEM107-GFP at the BB. (B) TMEM216-GFP transformants were treated with ControlRNAi, TMEM107RNAi, RPGRIP1LRNAi, and CEP290RNAi. ControlRNAi and TMEM216RNAi were used to quantify the efficiency of the silencing (see S2B Fig). Note that either CEP290RNAi, RPGRIP1LRNAi, or TMEM 216RNAi prevent the localization of TMEM216-GFP at BB. (C) CEP290-GFP transformants were treated with ControlRNAi, TMEM107RNAi, TMEM216RNAi, and RPGRIP1LRNAi. CEP290RNAi is used as a control. Note that the depletion of TMEM107, TMEM216, and RPGRIP1L do not modify the CEP290-GFP pattern, while depletion of CEP290 is greatly diminished, which demonstrates the silencing efficiency. (D) RPGRIP1L-GFP transformants were treated with ControlRNAi, TMEM107RNAi, TMEM216RNAi, and CEP290RNAi. RPGRIP1LRNAi is used as a control. Note that only CEP290RNAi prevents the localization of RPGRIP1L-GFP at the BB, while the depletion of TMEM107 and TMEM216 do not modify the RPGRIP1L-GFP pattern. See also the disappearance of the fluorescence after RPGRIP1LRNAi, which shows the silencing efficiency. Left panels: entire cell; right panels: magnification of BB rows. Bar: 10 μm. (TIF) [file pbio.3000640.s007.tif]
