# Supplementary material for: MKS-NPHP module proteins control ciliary shedding at the transition zone
Source: PLoS Biol. 2020 Mar 12;18(3):e3000640. doi: 10.1371/journal.pbio.3000640 (PMC7093003; doi:10.1371/journal.pbio.3000640)
Supplement: S1 Table — ID of TZ proteins in Paramecium. (PDF) [file pbio.3000640.s008.pdf]

Table S1: ID list of transition zone proteins in *Paramecium*

GENE NAME	GENE ID
MKS1	PTET.51.1.G0320263
MKS2/TMEM216	PTET.51.1.G0150351<br>PTETP2500008001<br>PTET.51.1.G0300269
MKS3/TMEM67/MECKELIN	PTET.51.1.G0460260
AHI1	PTET.51.1.G0800017<br>PTET.51.1.G1620035
B9D1	PTET.51.1.G1150027<br>PTET.51.1.G1130142
B9D2	PTET.51.1.G1470056<br>PTET.51.1.G0610100<br>PTET.51.1.G0280188
CC2D2A	PTET.51.1.G0190283
TECTONIC (1,2 and 3)	PTET.51.1.G0700187
TMEM17	PTET.51.1.P0500202<br>PTET.51.1.P1210132<br>PTET.51.1.P0380214
TMEM107	PTET.51.1.G0300250<br>PTET.51.1.P0310017
TMEM218	-
TMEM231	PTET.51.1.P1340134<br>PTET.51.1.P0010036
TMEM237	-
NPHP1	PTET.51.1.G5560037<br>PTET.51.1.G1530007
NPHP4/POC10	PTET.51.1.P0020231<br>PTET.51.1.G0220034<br>PTET.51.1.P0290127<br>PTET.51.1.P0130026
NPHP5/IQCB1	-
NPHP3/MKS7	PTET.51.1.P0280102<br>PTET.51.1.P0610178<br>PTET.51.1.G0340120<br>PTET.51.1.P0480171
CEP290/NPHP6/MKS4	PTET.51.1.G0130345<br>PTET.51.1.P0190305
RPGRIP1L/NPHP8	PTET.51.1.G0340252<br>PTET.51.1.G0480042
